# Fangchinoline suppresses conjunctival melanoma by directly binding FUBP2 and inhibiting the homologous recombination pathway

**DOI:** 10.1038/s41419-021-03653-4

**Published:** 2021-04-07

**Authors:** Keting Bao, Yongyun Li, Jinlian Wei, Ruoxi Li, Jie Yang, Jiahao Shi, Baoli Li, Jin Zhu, Fei Mao, Renbing Jia, Jian Li

**Affiliations:** 1 State Key Laboratory of Bioreactor Engineering, Shanghai Key Laboratory of New Drug Design, East China University of Science and Technology, 130 Mei Long Road, Shanghai, 200237 China; 2 Department of Ophthalmology, Ninth People’s Hospital, Shanghai Jiao Tong University School of Medicine, Shanghai, 200001 China; 3 College of Pharmacy and Chemistry, Dali University, 5 Xue Ren Road, Dali, Yunnan 671000 China; 4 Frontiers Science Center for Materiobiology and Dynamic Chemistry, East China University of Science and Technology, 130 Mei Long Road, Shanghai, 200237 China

**Keywords:** Tumour biomarkers, Target identification

## Abstract

Conjunctival melanoma (CM) is a rare and fatal ocular tumour with poor prognosis. There is an urgent need of effective therapeutic drugs against CM. Here, we reported the discovery of a novel potential therapeutic target for CM. Through phenotypic screening of our in-house library, fangchinoline was discovered to significantly inhibit the growth of CM cells including CM-AS16, CRMM1, CRMM2 and CM2005.1. Further mechanistic experiments indicated that fangchinoline suppressed the homologous recombination (HR)-directed DNA repair by binding with far upstream element binding protein 2 (FUBP2) and downregulating the expression of HR factors BRCA1 and RAD51. In vitro and in vivo antitumour experiments revealed that fangchinoline increased the efficacy of cisplatin by blocking HR factors and reduced the drug dose and toxicity. In conclusion, our work provides a promising therapeutic strategy for the treatment of CM that is worthy of extensive preclinical investigation.

## Introduction

Conjunctival melanoma (CM) is a rare and fatal ocular tumour that accounts for ~2% of all ocular malignancies^1^ and 1.6% of all non-cutaneous melanomas^2^. CM is more common in the Caucasian population (annual incidence of 0.2–0.5 per million^3^) compared to the Asian population (annual incidence of 0.15 per million^4^). It has previously been reported that CM emerges from the malignant transformation of neuroectoderm-derived melanocytes that migrate to the conjunctiva^5^ and may invade the orbit and eyeball, after which they metastasize to regional and systemic lymph nodes^6^. Currently, the first-line treatment for CM is wide local excision and biopsy with ‘no touch’ technique^7^, followed by adjunct treatment with cryotherapy, radiotherapy^8^ and chemotherapy using mitomycin C or interferon α-2b^9^. Among the Caucasian CM patients in a Dutch referral centre, the overall 5-year recurrence rate, metastasis rate and melanoma-related survival rate were reported to be 29%, 12% and 90%, respectively^10^. In another study on Chinese CM patients, the 10-year tumour-related recurrence rate, metastasis rate and mortality were observed to be 66%, 51% and 37%, respectively. The results of a large retrospective study on CM indicated that the Chinese CM patients presented a more compromised prognosis compared to the Caucasian CM patients, which might be attributed to the limited standard medical treatment available in rural areas, as well as the prevalence of varied tumour cell types among different races^11^.

Mutations in *BRAF* and *NRAS* are commonly observed in cutaneous melanoma, with incidences of 37%–50% and 13%–25%, respectively^12^. A previous study on 78 Caucasian cases reported that 29% of CM patients harboured *BRAF* mutation, whereas 18% harboured *NRAS* mutation^13^. However, another study on Chinese CM population revealed that *BRAF* mutation was detected in only 8% of the patients^14^, indicating that different races had different genetic mutation profiles. Notably, Caucasian patients with *NRAS*-mutant melanoma presented a more aggressive natural history and poorer survival rates than those with *BRAF*-mutant or wild-type melanoma^15,16^. To date, the mitogen-activated protein kinase (MEK) inhibitor, MEK162, is the only Food and Drug Administration-approved targeted drug used for the treatment of advanced cutaneous melanoma with *NRAS* mutation^17^. No *BRAF* or *NRAS* mutation-targeting drugs in CM patients have been approved, although some specific inhibitors have been shown to exhibit positive effects in clinical trials^18,19^. This has given rise to the need for developing novel therapeutic targets for CM patients.

Due to the unmet need, a phenotypic screening of our in-house 1347 marketed drugs against CM was conducted in CM-AS16 cells obtained from a Han Chinese patient with a typical *NRAS* mutation^20^ for potential candidate drugs. Our group focuses on drug repurposing and has achieved some success^21,22^. After an initial screening, cepharanthine was discovered to inhibit the proliferation of CM-AS16 cells. Meanwhile, its analogue fangchinoline showed comparable activity and was selected for further study. Fangchinoline inhibits the growth of various tumours^23^, but its anti-CM effect remains unknown. The transcriptome analysis demonstrated that fangchinoline suppressed the homologous recombination (HR) pathway in CM-AS16 cells. Furthermore, an activity-based protein profiling (ABPP) chemoproteomic approach was used to map the proteome-wide targets of fangchinoline in CM-AS16 cell proteome. It revealed FUBP2 to be one of the primary targets of fangchinoline. Furthermore, fangchinoline inhibited the expressions of downstream proteins of FUBP2, including c-Myc^24^, BRCA1 and RAD51^25^. All of these results may contribute to the development of novel CM-targeted therapies.

## Results

### Fangchinoline inhibited the growth of CM cells in vitro and in vivo

**Fig. 1:**
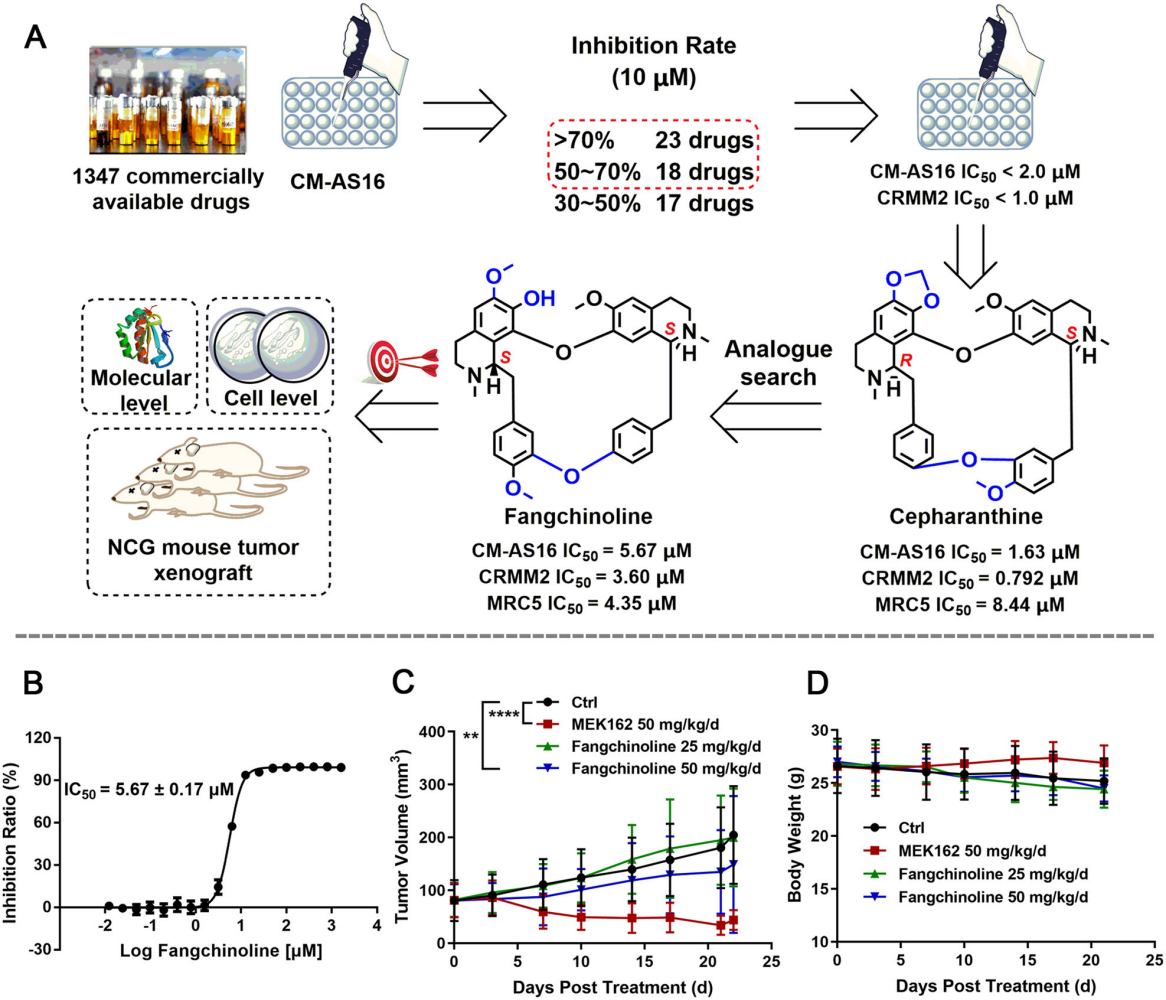
Therapeutic efficacy of fangchinoline in vitro and in vivo. **A** Schematic of the phenotypic screening protocol from which fangchinoline was discovered to be a potent inhibitor of CM-AS16 cell proliferation. **B** Inhibition curve of fangchinoline against CM-AS16 cells based on the CCK-8 assay. The results are shown as the mean ± SD (*n* = 3). **C** Tumour volume of CM-AS16 tumour xenografts in NCG mice. Mice were treated with increasing doses of fangchinoline (0, 25, and 50 mg/kg/d) or MEK162 (50 mg/kg/d) for 22 days (*n* = 7). Notably, three mice under 25 mg/kg fangchinoline treatment died before killing. After 22 days, the mice were killed and the tumours were removed and analysed. **D** Body weight changes in control and drug-treated NCG mice. Data represent the mean ± SD, *n* = 7 per group. *P < 0.05, **P < 0.01, ****P < 0.0001, by two-way ANOVA.

The anti-proliferative activity of our in-house drugs against CM-AS16 cells was assessed using the Cell Counting Kit-8 (CCK-8) assay at a final concentration of 10 μM (Fig. 1A). Of them, 41 drugs exhibited good inhibitory activity (inhibition rate > 50%) and were selected for further IC_50_ investigation in both CM-AS16 and another CM cell line CRMM2. Our results indicated that an approved natural drug cepharanthine significantly inhibited the growth of CM-AS16 cells with IC_50_ value of 1.63 μM. The other three cepharanthine analogues, berbamine hydrochloride, tetrandrine and fangchinoline, also inhibited CM-AS16 cell growth with IC_50_ values of 8.46, 3.20 and 5.67 μM, respectively (Supplementary Table S1). These analogues were Chinese herbal monomers harbouring the same bisbenzylisoquinoline core. Among them, fangchinoline exhibited a favourable structural plasticity (Fig. 1A, B). Fangchinoline harbours an exposed hydroxy group, which is suitable for synthesizing functionalized probes and subsequent target identification. Hence, it was selected for an in-depth investigation. Next, we assessed the anti-proliferative activity of fangchinoline against three other CM cell lines obtained from Caucasian patients with a typical *NRAS* (CRMM2) or *BRAF* mutation (CRMM1 and CM2005.1)^26,27^. Consistent with its potent antitumour activity against CM-AS16 cells, fangchinoline inhibited the cell proliferation in CRMM1, CRMM2 and CM2005.1 cells, with IC_50_ values of 2.68, 3.60 and 7.40 μM, respectively (Supplementary Table S1). To determine whether fangchinoline could suppress CM properties in vivo, an immunodeficient NCG mice xenograft model was created by subcutaneous inoculation of CM-AS16 cells. NCG mice comes from knocking out the *Prkdc* and *IL2rg* genes in NOD/ShiltJ mice through Crispr Cas9 technology. As shown in Fig. 1C, the CM-AS16 tumour growth was significantly inhibited after intraperitoneal (i.p.) treatment of the NCG male mice with fangchinoline at a dosage of 50 mg/kg/day compared to the control group (*p* < 0.01). The average tumour volume of the control group was 204 mm^3^, which was higher than that of the treated groups (50 mg/kg/day fangchinoline, tumour volume: 149 mm^3^ and 25 mg/kg/day fangchinoline, tumour volume: 200 mm^3^). The average tumour volume of group treated with 50 mg/kg/day fangchinoline decreased by about 27% compared to the control group, without any significant change in the average body weight (Fig. 1D). Similarly, there were no differences in the levels of alanine transaminase (ALT), aspartate transaminase (AST), creatinine or urea in mice serum of the fangchinoline-treated and control groups, indicating that fangchinoline does not have an impact on liver and renal functions (Supplementary Fig. S1).

### Fangchinoline suppressed the HR pathway in CM-AS16 cells

**Fig. 2:**
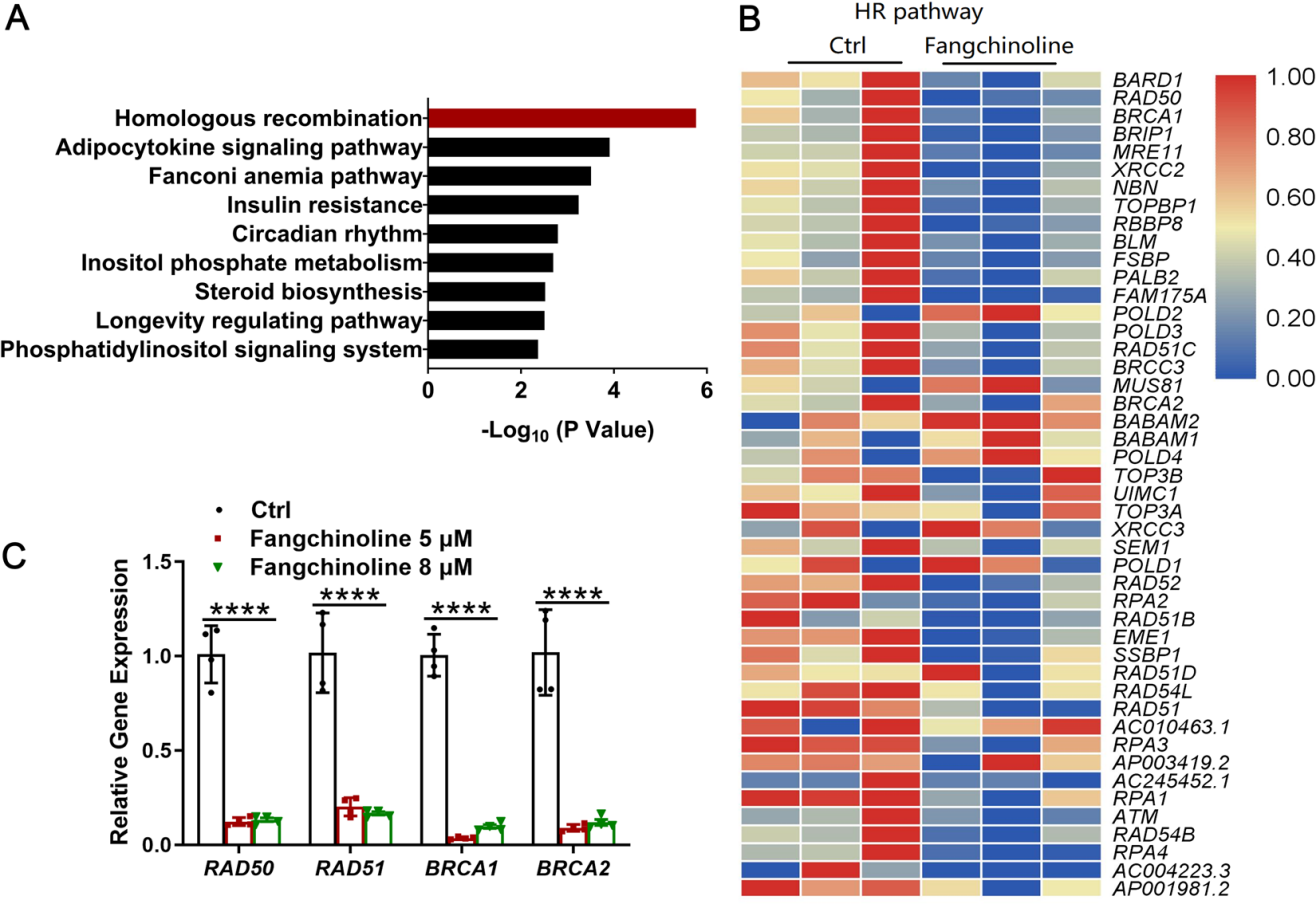
The effects of fangchinoline on the HR pathway in CM-AS16 cells were determined by transcriptome analysis and qRT-PCR. **A** KEGG enrichment analysis of DEGs altered by 8 μM fangchinoline. All pathways significantly enriched in the DEGs (adjusted *P* < 0.01) were included. **B** A heat map depicting DEGs altered by fangchinoline revealed a significant correlation with the HR pathway. The colour indicates the relative gene expression value. Blue: lowest expression; red: highest expression. **C** Quantification of the relative expression of genes related to the HR pathway. Data represent the mean ± SD of four independent experiments. *P < 0.05, ****P < 0.0001, by Student’s *t*-test.

To unravel the anti-proliferation mechanism of fangchinoline in CM-AS16 cells, transcriptome analysis was performed. As fangchinoline inhibited the cell proliferation of CM-AS16 cells with IC_50_ values within 5–8 μM, the concentration of 8 μM was selected for transcriptome analysis. To identify the differentially expressed genes (DEGs) between fangchinoline and control groups, the expression of each transcript was calculated according to the fragments per kilobase of exon per million mapped reads method. The Kyoto encyclopedia of genes and genomes (KEGG) enrichment was performed via Fisher’s exact test using KOBAS software and was performed to identify which DEGs were enriched. It revealed that nine signalling pathways were significantly affected by fangchinoline treatment (adjusted *P* < 0.01) and the top-ranking functional cluster was the HR pathway, with an adjusted *P*-value < 1 × 10^−5^ (Fig. 2A). HR is a critical cellular process that contributes to DNA repair and DNA damage response (DDR). When exposed to genotoxic stresses, cells use the HR pathway to repair DNA double-stranded breaks (DSBs) and interstrand crosslinks (ICLs) with high fidelity^28^. HR is generally mediated by a repertoire of conserved factors, including the essential recombinase, RAD51. Cells deficient in RAD51 are unviable due to its indispensable role in the HR pathway^29^.

Transcriptome analysis of fangchinoline-treated cells suggested significant changes at the transcriptional level in genes associated with the DNA repair and DDR pathways, including the HR pathway (Fig. 2B). Subsequently, we used quantitative real-time PCR (qRT-PCR) to detect the expression of some important genes involved in the HR pathway: *BRCA1*, *BRCA2*, *RAD50* and *RAD51*. In line with these results, we observed that these genes were significantly downregulated in CM-AS16 cells treated with 5 and 8 μM fangchinoline (Fig. 2C). Taken together, fangchinoline was responsible for HR suppression in CM-AS16 cells.

### Fangchinoline targets FUBP2 in CM-AS16 cells

**Fig. 3:**
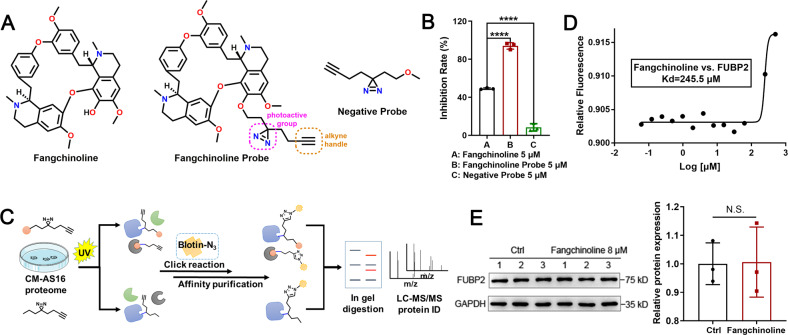
Target search and verification of fangchinoline. **A** Chemical structures of fangchinoline, the fangchinoline probe and the negative probe. The diazirine photo-cross-linking group is circled in pink, and the alkyne reporter group is circled in orange. **B** The inhibition rates of fangchinoline, fangchinoline probe and negative probe at a concentration of 5 μM against CM-AS16 cells. *n* = 3. **C** Schematic representation of the ABPP assay used to identify targets of fangchinoline in CM-AS16 cells. The ‘orange ball’ represents fangchinoline. **D** MST analysis of the binding affinity between fangchinoline and FUBP2. The measured *K*_d_ value is shown. **E** Immunoblotting for FUBP2 (left) and quantification of its protein abundance (right) in CM-AS16 cells with or without 8 μM fangchinoline treatment. Data represent the mean ± SD of three independent experiments. *P < 0.05, ****P < 0.0001, by Student’s *t*-test.

Although fangchinoline exhibited anti-proliferative properties against many types of cancer cells^23^ including CM cells (as shown in this study), its direct targets are still undefined. To explore the mechanisms of fangchinoline, ABPP assay was conducted. ABPP is one of the chemical proteomic approaches that use small-molecule chemical probes to elucidate the interactions between compounds and targets, which is widely used for the discovery of functional targets of bioactive compounds^30^. First, we designed and synthesized a photoaffinity fangchinoline probe that contained a diazirine photo-cross-linking group and a ‘clickable’ handle consisting of an alkynyl group (Fig. 3A and Supplementary Chemical Synthesis). This fangchinoline probe retained its original anti-proliferative activity against CM-AS16, CRMM1, CRMM2 and CM2005.1 cells, with IC_50_ values of 0.95, 1.90, 4.26 and 3.68 μM, respectively (Supplementary Table S1). The negative probe had no effect on the growth of CM-AS16 cells with an inhibition rate below 10% under the concentration of 5 μM (Fig. 3B). The overall scheme of the ABPP experiments is shown in Fig. 3C. Briefly, the probes were separately incubated in CM-AS16 cell lysates, followed by ultraviolet (UV)-induced photo-cross-linking. Next, the probe-labelled mixture was conjugated with azide-biotin via click chemistry and enriched on streptavidin columns. Finally, nine proteins were collectively identified as potential targets that specifically bound to fangchinoline (Supplementary Table S2). As fangchinoline effectively blocked the HR pathway, FUBP2 was selected because of its crucial role in DNA repair and DDR. FUBP2 is a multifunctional nucleic acid-binding protein belonging to the FUBP family. It plays an important role in cancer invasion and has been identified as a metastasis-associated candidate molecule. In addition, this protein controls the transcription of proto-oncogenes such as *c-Myc*^31^ (which is overexpressed in more than half of human cancers) and is associated with tumour initiation, progression and maintenance^32^. As a transcription factor, c-Myc directly upregulates the HR factors, BRCA1 and RAD51^33,34^.

A microscale thermophoresis (MST) assay showed that fangchinoline directly bound to FUBP2 with an equilibrium dissociation constant (*K*_d_) of 245.5 μM (Fig. 3D). In addition, the immunoblotting assays showed that treatment with 8 μM fangchinoline did not impact the FUBP2 protein level in CM-AS16 cells (Fig. 3E). These data demonstrated that fangchinoline bound to FUBP2 without affecting its expression.

### Fangchinoline suppressed the HR pathway and increased the sensitivity of CM-AS16 cells to DNA damage-inducing drugs

**Fig. 4:**
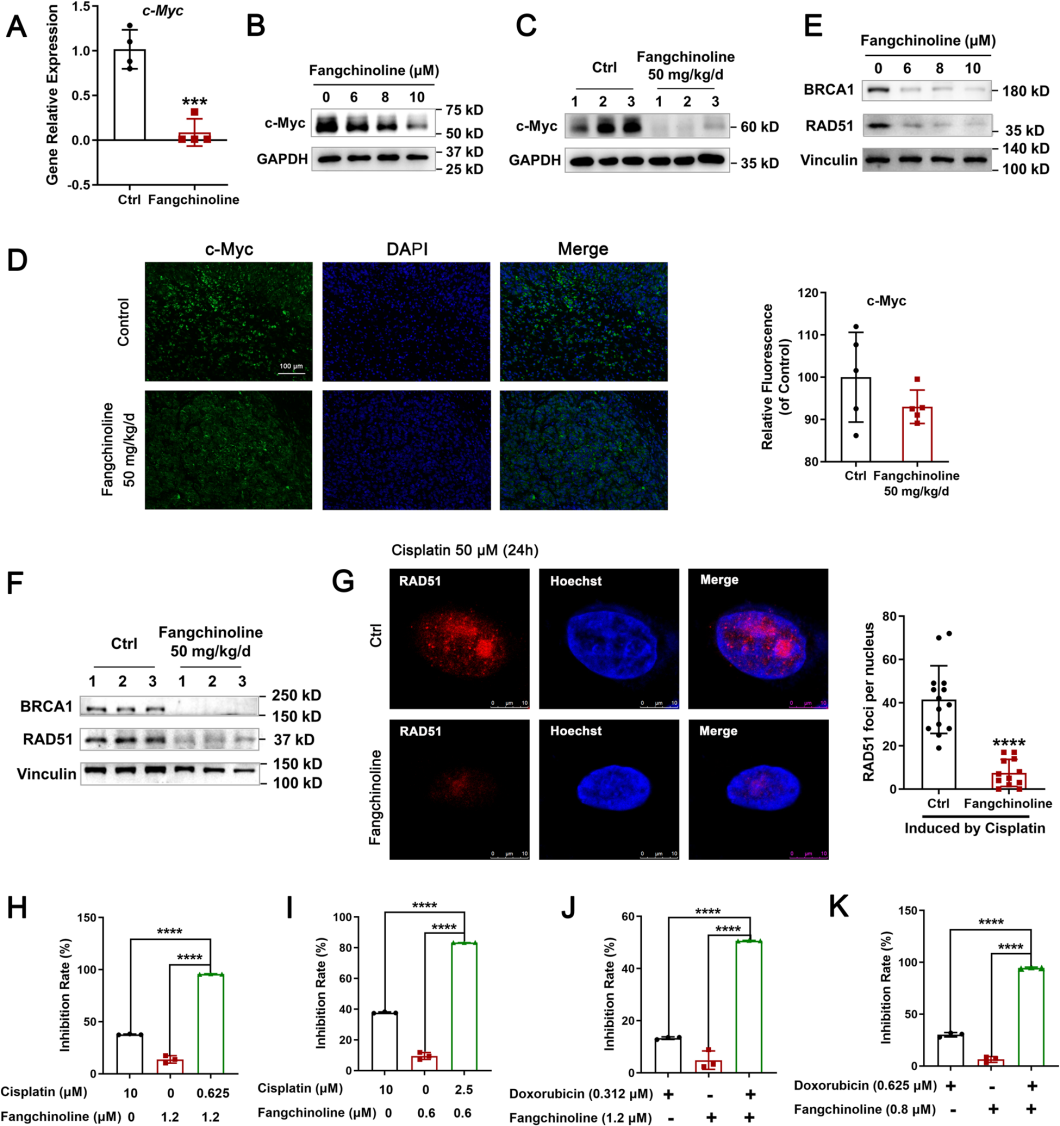
Fangchinoline suppressed the HR pathway and increased sensitivity to DNA damage-inducing drugs. **A** Transcriptional expression of *c-Myc* was decreased in CM-AS16 cells after treatment with 8 μM fangchinoline. *n* = 4. **B** The protein level of c-Myc was decreased in a dose-dependent manner in CM-AS16 cells after treatment with fangchinoline. *n* = 3. **C** An immunoblotting assay revealed that the protein level of c-Myc was decreased in CM-AS16 tumour tissues after treatment with 50 mg/kg fangchinoline daily. *n* = 3. **D** An immunofluorescent assay revealed that the protein level of c-Myc was decreased in CM-AS16 tumour tissues after treatment with 50 mg/kg fangchinoline daily. Data are shown as the mean ± SD, *n* = 5. Scale bar: 100 μm. **E** The protein levels of the HR factors BRCA1 and RAD51 were decreased in a dose-dependent manner in CM-AS16 cells after treatment with fangchinoline. *n* = 3. **F** The protein levels of the HR factors BRCA1 and RAD51 were decreased in CM-AS16 tumour tissues after treatment with 50 mg/kg fangchinoline daily. *n* = 3. **G** Representative images of RAD51 foci induced by cisplatin (50 μM for 24 h) in CM-AS16 cells after treatment with or without 1.2 μM fangchinoline [red: RAD51; blue: Hoechst]. Scale bar: 10 μm. Fangchinoline significantly decreased the number of RAD51 foci induced by cisplatin. *n* = 3. **H**, **I** Fangchinoline increased the sensitivity of CM-AS16 cells to cisplatin. **J**, **K** Fangchinoline increased the sensitivity of CM-AS16 cells to doxorubicin. *n* = 3. Data represent the mean ± SD of three independent experiments. *P < 0.05, ***P < 0.001, ****P < 0.0001, by Student’s *t*-test.

As FUBP2 upregulated *c-Myc* transcriptional level, we first evaluated the expression of *c-Myc* after fangchinoline treatment in CM-AS16 cells. As shown in Fig. 4A, the mRNA level of *c-Myc* sharply declined after treatment with 8 µM fangchinoline in CM-AS16 cells. Meanwhile, a significant dose-dependent decrease in c-Myc protein level was observed in fangchinoline-treated CM-AS16 cells (Fig. 4B). Furthermore, as expected, immunoblotting and immunofluorescent assays of the CM-AS16 grafts also showed a decrease in c-Myc level after administration of 50 mg/kg/day fangchinoline (Fig. 4C, D). As *BRCA1* and *RAD51* are transcriptionally regulated by c-Myc, we evaluated the effect of fangchinoline on their expression in CM-AS16 cells. Consistent with the results from previous qRT-PCR analysis, BRCA1 and RAD51 expressions were inhibited at both transcriptional and protein levels (Figs. 2C and 4E). Meanwhile, the CM-AS16 grafts also showed that the levels of BRCA1 and RAD51 decreased after administration of 50 mg/kg/day fangchinoline (Fig. 4F).

Platinum‐based agents have been widely used to treat cancers by blocking DNA replication and transcription, as they can lead to generation of various types of DNA adducts, such as ICLs^35,36^. However, the cells have certain repair mechanisms to resolve these lesions. The HR pathway allows the cells to cope with genotoxic stresses by repairing DNA DSBs and ICLs, which explains why BRCA1/2-deficient cancer cells are hypersensitive to platinum compounds^28,37^. BRCA1 and RAD51 play a central role in HR-mediated DSB repair^38,39^ and mediate the response of tumour cells to cisplatin^40,41^. As fangchinoline substantially decreased BRCA1 and RAD51 levels, we further assessed whether combined administration of fangchinoline and platinum compounds could be used as a safe and effective therapy.

As the formation of RAD51 foci is regarded as a critical step in HR, we determined whether the formation of these foci is impaired by fangchinoline^42^. As shown in Fig. 4G, treatment with fangchinoline for 24 h significantly attenuated the number of RAD51 foci in CM-AS16 cells, which were induced by treatment with cisplatin for 24 h. This result further supported our finding that fangchinoline functionally suppressed the HR pathway in CM cells.

To further confirm these results, we assessed the combined effect of fangchinoline and cisplatin in CM-AS16 cells. CM-AS16 cells were treated with different concentrations of cisplatin in the presence of 0.6 μM or 1.2 μM fangchinoline. As shown in Fig. 4H, I, both cisplatin (10 μM) and fangchinoline (0.6 or 1.2 μM) showed <50% inhibition when applied alone. However, combination of these two drugs lead to a significant synergistic effect. In addition, the combination of fangchinoline with another DNA damage-inducing drug doxorubicin also led to a significant synergistic effect (Fig. 4J, K), which indicated that fangchinoline treatment increased the sensitivity of CM-AS16 cells to cisplatin and doxorubicin by interfering with DNA repair and promoting DNA damage.

We next examined the in vivo antitumour effects of fangchinoline, cisplatin and their combination. As shown in Supplementary Fig S9A, in A375 cells, fangchinoline downregulated the expressions of c-Myc, BRCA1 and RAD51. Meanwhile, in consideration of the limited growth rate of CM-AS16 cells, we chose A375 cutaneous melanoma cells for further research. As shown in Supplementary Fig. S2a, the potency of a combination of fangchinoline and cisplatin was significantly greater than that of fangchinoline or cisplatin alone. Taken together, our results suggested that fangchinoline increased the in vivo antitumour efficacy of cisplatin and reduced its dosage and toxicity. Moreover, we observed that fangchinoline downregulated the expression of c-Myc in the A375 tumour tissues (Supplementary Fig. S3), which indicated that fangchinoline and cisplatin acted synergistically to induce a higher degree of DNA damage. Furthermore, combined fangchinoline and cisplatin treatment increased the proportion of terminal deoxynucleotidyl transferase‐mediated dUTP biotin nick end labelling (Tunel)-positive cells (Supplementary Fig. S2b) compared to tissues treated with either fangchinoline or cisplatin, which indicated that fangchinoline conferred increased sensitivity to cisplatin.

### FUBP2 facilitated the HR pathway

**Fig. 5:**
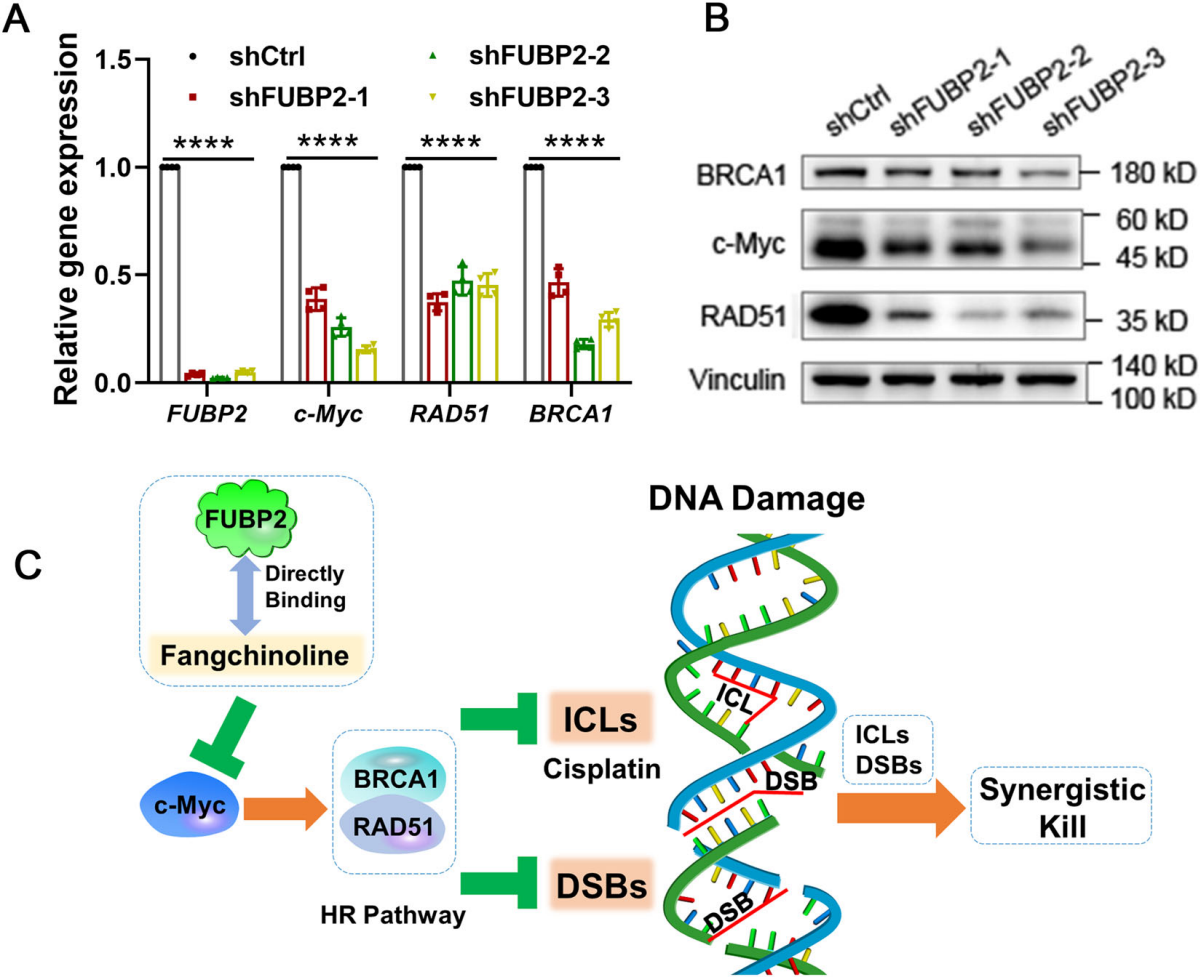
Effects of FUBP2 on the HR pathway. **A** The transcriptional expressions of *FUBP2*, *c-Myc*, *RAD51* and *BRCA1* were reduced after treatment with shFUBP2s in CM-AS16 cells. *n* = 4. **B** The protein expressions of c-Myc, RAD51 and BRCA1 were decreased in CM-AS16 cells after treatment with shFUBP2s. *n* = 3. **C** Schematic diagram of the action of fangchinoline in regulating the HR pathway. Data represent the mean ± SD of at least three independent experiments. *P < 0.05, ****P < 0.0001, by two-way ANOVA.

To investigate the role of FUBP2 in HR pathway, genetic silencing experiments were conducted. As shown in Fig. 5A, B, FUBP2 knockdown significantly decreased the expression of c-Myc and HR factors (BRCA1 and RAD51) at both the mRNA and protein level in CM-AS16 cells, which suggested that FUBP2 silencing suppressed HR-directed DNA repair and lead to DNA damage. Overall, the results indicated that fangchinoline significantly inhibited the HR pathway by binding to FUBP2 and downregulating the expressions of c-Myc, BRCA1, and RAD51. Thus, fangchinoline could potentially increase the cellular sensitivity to DNA damage-inducing drugs and induce synergistic killing effect (Fig. 5C).

### Fangchinoline suppressed the HR pathway and increased the sensitivity of other CM cells to DNA damage-inducing drugs

To explore whether the effects of fangchinoline treatment in CM-AS16 cells is applicable in the other CM cells too, CM2005.1, CRMM1 and CRMM2 cells were treated with fangchinoline or shFUBP2. As shown in Supplementary Figs. S6A–C, G, H, S7A–C, G, H and S8A–C, G, H, fangchinoline or shFUBP2 treatment in the other three CM cells reduced the expressions of c-Myc, RAD51 and BRCA1 at both the mRNA and protein levels by binding to FUBP2. Moreover, as shown in Supplementary Figs. S6D–F, S7D–F and S8D–F, fangchinoline significantly reduced the formation of cisplatin-induced RAD51 foci and increased the sensitivity to cisplatin and doxorubicin in the other three CM cells too.

## Discussion

In recent years, several signalling pathways, such as the FAK, MEK-ERK1/2, NF-κB, and PI3K-Akt-mTOR, have been proposed as the targets of fangchinoline owing to its anticancer activity, which suggested the broad activity spectrum of this herbal monomer in different tumour cells^23^. Herein, our results showed that fangchinoline inhibited the proliferation of CM-AS16 cells both in vitro and in vivo. Notably, this compound reduced the expression of c-Myc at both the mRNA and protein levels by binding to FUBP2, which is also a potential therapeutic target for anti-melanoma^43^. Furthermore, fangchinoline downregulated the expressions of BRCA1 and RAD51 in CM-AS16 cells, which were the downstream effectors of c-Myc in the HR pathway. In addition, fangchinoline inhibited the growth of other types of CM cells, including CM2005.1, CRMM1 and CRMM2 cells, through the same mechanism (Supplementary Figs. S6A–C, S7A–C and S8A–C). Moreover, the efficacy of Fangchinoline-FUBP2-c-Myc-HR axis was not only observed in CM cells but has also been found to be crucial in other types of cancer cells such as A375, K562, A549, ISK and SKOV3 (Supplementary Fig. S9).

Cisplatin-resistant cells exhibit an elevated DNA-repair ability^44^, which, in turn, increases the survival of cancer cells. Therefore, the development of small molecules that modulate or disrupt the HR pathway has recently attracted increasing attention^45,46^. In our study, we observed that fangchinoline significantly reduced the formation of cisplatin-induced RAD51 foci and inhibited the proliferation of CM-AS16 cells by impairing DNA repair. Thus, fangchinoline could potentially be used as a sensitizing agent in chemotherapy. Similar effects have been observed for other types of CM cells, including CM2005.1, CRMM1 and CRMM2 cells (Supplementary Figs. S6D–F, S7D–F and S8D–F). As expected, fangchinoline significantly downregulated the levels of c-Myc, BRCA1 and RAD51, both in vitro and in vivo, increased the sensitivity to cisplatin and decreased the drug dosage and toxicity, which could be used to overcome cisplatin resistance.

In conclusion, our study revealed a novel and unique mechanism of fangchinoline that leads to the suppression of CM. Fangchinoline can be used as an adjuvant drug in the therapy of CM or other kinds of cancer and exhibits great potential as a candidate drug in combination therapies.

## Materials and methods

### Reagents

Fangchinoline (98%) was from Chengdu DeSiTe Biological Technology Co., Ltd (DF0005, Chengdu, China). Cepharanthine, berbamine hydrochloride and tetrandrine (98%) were from Bidepharm. MEK162 was from CSNpharm (CSN16001, Chicago, USA). Dulbecco’s modified Eagle medium (DMEM) and phosphate buffer solution (PBS) were from Hyclone. Fetal bovine serum (FBS, 42F6590K), RPMI-1640 medium HEPES (1640H, 22400089) and Ham’s F12K (Kaighn’s) medium (F12K, 21127022) were from Gibco (Grand Island, NY, USA). Multicolour protein markers were from Absin Bioscience, Inc. (abs923, Shanghai, China) and Yeasen (20352ES76, Shanghai, China).

### Cell culture

CRMM1, CRMM2 and CM2005.1 were generously provided by Professor Martine J. Jager (Leiden University Medical Center, Leiden, The Netherlands)^47^ and CM-AS16 was provided by Professor Renbin Jia (Shanghai Ninth People’s Hospital, Shanghai, China). A375 and HEK293T were obtained from American Type Culture Collection. CM-AS16 and CM2005.1 were maintained in 1640H medium, CRMM1 and CRMM2 were maintained in F12K medium, A375 and HEK293T were maintained in DMEM medium, and MRC5 was maintained in MEM medium. All mediums were supplemented with 10% FBS. Cells were cultured at 37 °C in an incubator, in a humidified atmosphere with 5% CO_2_, periodically checked for mycoplasma-free and validated by short tandem repeats (STR) DNA analysis at BGI Tech Solutions (Beijing Liuhe) Co., Limited.

### Cell viability assay

Cells (1 × 10^4^ cells/well) were seeded in 96-well plates (100 μL/well). In succession, compounds in 100 μL fresh medium were added and incubated for 72 h. After removal of the cell culture medium, a 10% CCK-8 (Apexbio, K1018, Houston, USA) solution in medium was administered and re-incubated for 2 h. The absorbance at 450 nm was measured in a microplate reader (Biotek, Vermont, USA).

### Transcriptome analysis by RNA-sequencing

CM-AS16 cells were treated with or without 8 μM fangchinoline. The transcriptome analysis by RNA-sequencing was performed according to a previously published method^48^. The technique we used was the second-generation sequencing technology. Total RNAs were extracted using Trizol Reagent (R0016, Beyotime, Shanghai, China). Further assay and analysis were assisted by Majorbio Bio-Pharm Technology Co. Ltd (Shanghai, China) and are shown in Supplementary Dataset S1.

### ABPP assay

CM-AS16 cells were lysed, incubated with probes and radiated with UV. Then, the mixtures were combined with 300 µM biotin-azide, 100 µM TBTA, 1 mM TCEP and 1 mM CuSO_4_. After that, the proteome was performed as the protocol of Capturem Streptavidin Miniprep Columns (635733, Takara, Beijing, China), separated by SDS-polyacrylamide gel electrophoresis and visualized by Coomassie blue staining. The protein bands in the whole gel were excised, followed by in-gel digestion and analysis by liquid chromatography-tandem mass spectrometry at Majorbio Bio-Pharm Technology Co. Ltd (Shanghai, China) and are shown in Supplementary Dataset S2.

### Cell transfection

Taitool Bioscience (Shanghai, China) assisted in preparing FUBP2 short hairpin RNA adenovirus.

The target sequences of FUBP2 are designed as follows:

shFUBP2-1, sense: 5′-CAAGATGATGCTGGATGACAT-3′;

shFUBP2-2, sense: 5′-GATGATCAAGAAGATCCAGAA-3′;

shFUBP2-3, sense: 5′-GATCATCAACGACCTCCTCCA-3′;

shControl, sense: 5′-AAATGTACTGCGCGTGGAGAC-3′.

Cell infection was performed by direct addition of adenovirus particles to pelleted cells for 24 h and transduced cells were collected 24 h post infection for the qRT-PCR and western blot assay.

HEK293T cells were transfected with plasmids (EX-Z9878-M93, GeneCopoeia, Inc., Rockville, MD, USA) with Lipofectamine 3000 (L3000015, Invitrogen, Carlsbad, CA, USA). The transfection duration was 72 h.

### Antibodies

Primary antibodies were against FUBP2 (ab150393, Abcam), c-Myc (ab32072, Abcam), RAD51 (ab133534, Abcam), BRAC1 (YT0519, ImmunoWay), GAPDH (60004-1-lg, Proteintech) and Vinculin (ab129002, Abcam).

Secondary antibodies were Cy3-labelled goat anti-rabbit IgG (H + L) (A-11035, Invitrogen), Alexa 488-conjugated anti-rabbit IgG (GB25303, Servicebio), HRP-linked Anti-rabbit IgG Antibody (7074 S, Cell Signaling Technology) and HRP-linked Anti-mouse IgG Antibody (7076 S, Cell Signaling Technology).

### MST assay

An MST assay was conducted to test the binding affinity of fangchinoline and FUBP2 by using Monolith NT.115 (NanoTemper Technologies). FUBP2 labelled with enhanced green fluorescent protein was obtained from HEK293T cells transfected with plasmids and was used at a concentration of 10 μM. Fangchinoline was titrated in 1 : 1 dilutions beginning at a concentration of 500 μM in 5% (v/v) dimethyl sulfoxide (DMSO). Samples were diluted in MST assay buffer containing 25 mM HEPES (pH 7.0/7.5) supplemented with 5% (v/v) DMSO. For the measurement, the samples were filled into premium coated capillaries.

### Immunofluorescence staining

Cells were pre-treated with cisplatin for 24 h. After that, cisplatin was removed and cells were treated with fangchinoline for 24 h. Cells were fixed with 4% paraformaldehyde (PFA) and blocked with 5% bovine serum albumin containing 0.3% Triton X-100, then incubated with primary and secondary antibody. Hoechst 33258 (KGA211-10, KeyGEN BioTECH Co., Ltd, Nanjing, China) staining was performed before observation. Confocal fluorescence images were measured by a Leica confocal microscope (Leica TCS SPS CFSMP, Germany).

### Western blotting and qRT-PCR analysis

Cells were treated with fangchinoline for 24 h. The western blotting and qRT-PCR analysis protocols were used as described previously^49^. The sequences of primers are listed in Supplementary Information.

### Tissue immunofluorescence and Tunel assays

The tissue immunofluorescence^50^ and Tunel assay^51^ protocols were used as described previously.

### Mouse tumour xenograft studies

Animal experiments were carried out according to the National Institutes of Health guidelines and the Association for Research in Vision and Ophthalmology guidelines.

NCG mouse tumour xenograft experiments were conducted with the approval of the Institutional Animal Care and Use Committee (IACUC) of GemPharmatech Co. Ltd (Nanjing, Jiangsu, China) (Permit Number: SYXK (Jiangsu) 2018-0027; Animal Protocol No: GPTAP016). CM-AS16 cells (1.0 × 10^7^ in 100 μL of PBS) were injected subcutaneously into male NCG mice (6–7 weeks old). When the tumours reached a volume of ~80 mm^3^, the mice were randomized into treatment and control groups (seven mice per group). Mice in treatment groups received MEK162 (50 mg/kg, intragastric) or fangchinoline (25 mg/kg and 50 mg/kg, i.p.) once a day for 22 days. Three mice in the 25 mg/kg fangchinoline treatment group died before the end point, which might be due to the metastasis in the thymus, or weight loss or tumour enlargement.

Nu/nu nude mouse tumour xenograft experiments were reviewed and approved by the IACUC of the Shanghai Ninth People’s Hospital Central Laboratory (Permit Number: SYXK (Shanghai) 2016-0016). A375 cells (3.0 × 10^6^ in 100 μL of PBS) were subcutaneously inoculated into male nu/nu nude mice (5–6 weeks old) (Shanghai Jiesijie Laboratory Animal Co., Ltd). When the tumours reached a volume of ~160 mm^3^, the mice were randomized into treatment and control groups (*n* = 6 per group). Mice in treatment groups received fangchinoline (10 mg/kg and 50 mg/kg, i.p.), cisplatin (2 mg/kg, i.p.) or both once a day for 14 days.

The tumour size and body weight were monitored every 3 or 4 days. Tumour volume was calculated as 0.5 × length × width^2^. Then, the animals were killed by CO_2_ and the tumour xenografts were removed and photographed. Half of each tumour tissue was used for western blotting and the other half was fixed with 4% PFA for immunofluorescence staining. The blood samples were collected from submandibular venous plexus in NCG mice after being anesthesized with CO_2_.

### Statistical analysis

All data are represented as mean ± SD. Statistical analysis was conducted by Graphpad Prism 7.04 and significant differences within treatments were determined by two-way analysis of variance or Student’s *t*-test. *P* ≤ 0.05 was considered statistically significant.

## Supplementary information

Supplementary Figure Legends

Supplementary Chemical Synthesis

Supplementary Information

Supplementary Table 1

Supplementary Table 2

Figure S1. Effects of fangchinoline on liver and kidney damage in CM-AS16 xenograft tumor model.

Figure S2. Fangchinoline increased the sensitivity of A375 cells to cisplatin on nu/nu nude mice.

Figure S3. Fangchinoline decreased the levels of c-Myc compared to controls in A375 tumor tissues.

Figure S4. The structures of cepharanthine, fangchinoline, tetrandrine and berbamine hydrochloride.

Figure S5. The untrimmed whole western blot images in the manuscript.

Figure S6. Fangchinoline suppressed the HR pathway and increased sensitivity to DNA damage-inducing drugs in CM2005.1 cells.

Figure S7. Fangchinoline suppressed the HR pathway and increased sensitivity to DNA damage-inducing drugs in CRMM1 cells.

Figure S8. Fangchinoline suppressed the HR pathway and increased sensitivity to DNA damage-inducing drugs in CRMM2 cells.

Figure S9. Fangchinoline suppressed the HR pathway.

Figure S10. The untrimmed whole western blot images in the supporting information Figure S6, Figure S7 and Figure S8.

Figure S11. The untrimmed whole western blot images in the supporting information Figure S9.

Figure S12. The chemical synthesis of positive and negative probes.

Dataset S1

Dataset S2

## Data Availability

The accession number for RNA-seq reported in this paper is GEO: GSE164340.

## References

[1] McCartney AC (1995). Pathology of ocular melanomas. Br. Med. Bull..

[2] Scotto J, Fraumeni JF, Lee JA (1976). Melanomas of the eye and other noncutaneous sites: epidemiologic aspects. J. Natl Cancer Inst..

[3] Vora GK, Demirci H, Marr B, Mruthyunjaya P (2017). Advances in the management of conjunctival melanoma. Surv. Ophthalmol..

[4] Hu DN, Yu G, McCormick SA, Finger PT (2008). Population-based incidence of conjunctival melanoma in various races and ethnic groups and comparison with other melanomas. Am. J. Ophthalmol..

[5] Tolleson WH (2005). Human melanocyte biology, toxicology, and pathology. J. Environ. Sci. Health C. Environ. Carcinog. Ecotoxicol. Rev..

[6] Nair BCJ (2011). Conjunctival melanoma: bladder and upper urinary tract metastases. J. Clin. Oncol..

[7] Shields JA, Shields CL, De Potter P (1997). Surgical management of conjunctival tumors. Arch. Ophthalmol..

[8] Burattini S, Battistelli M, Falcieri E (2010). Morpho-functional features of in-vitro cell death induced by physical agents. Curr. Pharm. Des..

[9] Fernandes BF (2014). Local chemotherapeutic agents for the treatment of ocular malignancies. Surv. Ophthalmol..

[10] Brouwer NJ (2018). Treatment of conjunctival melanoma in a Dutch referral centre. Br. J. Ophthalmol..

[11] Zhou CD, Wang YX, Jia RB, Fan XQ (2017). Conjunctival melanoma in Chinese patients: local recurrence, metastasis, mortality, and comparisons with Caucasian patients. Invest. Ophthalmol. Vis. Sci..

[12] Sarkisian S, Davar D (2018). MEK inhibitors for the treatment of NRAS mutant melanoma. Drug Des. Devel. Ther..

[13] Lake SL (2011). Multiplex ligation-dependent probe amplification of conjunctival melanoma reveals common BRAF V600E gene mutation and gene copy number changes. Invest. Ophthalmol. Vis. Sci..

[14] Griewank KG (2013). Conjunctival melanomas harbor BRAF and NRAS mutations and copy number changes similar to cutaneous and mucosal melanomas. Clin. Cancer Res..

[15] Jakob JA (2012). NRAS mutation status is an independent prognostic factor in metastatic melanoma. Cancer.

[16] Thomas NE (2015). Association between NRAS and BRAF mutational status and melanoma-specific survival among patients with higher-risk primary melanoma. JAMA Oncol..

[17] Queirolo P, Spagnolo F (2017). Binimetinib for the treatment of NRAS-mutant melanoma. Expert Rev. Anticancer Ther..

[18] Maleka A, Åström G, Byström P, Ullenhag GJ (2016). A case report of a patient with metastatic ocular melanoma who experienced a response to treatment with the BRAF inhibitor vemurafenib. BMC Cancer.

[19] Mor JM, Heindl LM (2017). Systemic BRAF/MEK inhibitors as a potential treatment option in metastatic conjunctival melanoma. Ocul. Oncol. Pathol..

[20] Li Y (2018). Characterization of a conjunctival melanoma cell line CM-AS16, newly-established from a metastatic Han Chinese patient. Exp. Eye Res..

[21] Chen F (2016). Small-molecule targeting of a diapophytoene desaturase inhibits S. aureus virulence. Nat. Chem. Biol..

[22] Wang Y (2016). Discovery of potent Benzofuran-derived diapophytoene desaturase (CrtN) inhibitors with enhanced oral bioavailability for the treatment of methicillin-resistant *Staphylococcus aureus* (MRSA) infections. J. Med. Chem..

[23] Mérarchi M (2018). Molecular targets modulated by fangchinoline in tumor cells and preclinical models. Molecules.

[24] Avigan MI, Strober B, Levens D (1990). A far upstream element stimulates c-myc expression in undifferentiated leukemia cells. J. Biol. Chem..

[25] Luoto KR (2010). Tumor cell kill by c-MYC depletion: role of MYC-regulated genes that control DNA double-strand break repair. Cancer Res..

[26] Nareyeck G, Wuestemeyer H, von der Haar D, Anastassiou G (2005). Establishment of two cell lines derived from conjunctival melanomas. Exp. Eye Res..

[27] Keijser S, Maat W, Missotten GS, de Keizer RJ (2007). A new cell line from a recurrent conjunctival melanoma. Br. J. Ophthalmol..

[28] Hosoya N, Miyagawa K (2014). Targeting DNA damage response in cancer therapy. Cancer Sci..

[29] Bhattacharyya A, Ear US, Koller BH, Weichselbaum RR, Bishop DK (2000). The breast cancer susceptibility gene BRCA1 is required for subnuclear assembly of Rad51 and survival following treatment with the DNA cross-linking agent cisplatin. J. Biol. Chem..

[30] Cravatt BF, Wright AT, Kozarich JW (2008). Activity-based protein profiling: from enzyme chemistry to proteomicchemistry. Annu. Rev. Biochem..

[31] He L (2000). Loss of FBP function arrests cellular proliferation and extinguishes c-myc expression. EMBO J..

[32] Gabay M, Li Y, Felsher DW (2014). MYC activation is a hallmark of cancer initiation and maintenance. Cold Spring Harb. Perspect. Med..

[33] Carey JPW (2018). Synthetic lethality of PARP inhibitors in combination with MYC blockade is independent of BRCA status in triple-negative breast cancer. Cancer Res..

[34] Li M, Li A, Zhou S, Lv H, Yang W (2019). SPAG5 upregulation contributes to enhanced c-MYC transcriptional activity via interaction with c-MYC binding protein in triple-negative breast cancer. J. Hematol. Oncol..

[35] Niedernhofer LJ, Lalai AS, Hoeijmakers JHJ (2005). Fanconi anemia (cross)linked to DNA repair. Cell.

[36] Duxin JP, Walter JC (2015). What is the DNA repair defect underlying Fanconi anemia?. Curr. Opin. Cell Biol..

[37] Lohse I (2015). BRCA1 and BRCA2 mutations sensitize to chemotherapy in patient‐derived pancreatic cancer xenografts. Br. J. Cancer.

[38] Moynahan ME, Chiu JW, Koller BH, Jasin M (1999). Brca1 controls homology-directed DNA repair. Mol. Cell.

[39] Ledermann JA, Drew Y, Kristeleit RS (2016). Homologous recombination deficiency and ovarian cancer. Eur. J. Cancer.

[40] Takenaka T (2007). Combined evaluation of Rad51 and ERCC1 expressions for sensitivity to platinum agents in non-small cell lung cancer. Int. J. Cancer.

[41] Xiao M (2017). Let-7e sensitizes epithelial ovarian cancer to cisplatin through repressing DNA double strand break repair. J. Ovarian Res..

[42] Wang B (2015). Artesunate sensitizes ovarian cancer cells to cisplatin by downregulating RAD51. Cancer Biol. Ther..

[43] Liu W (2019). KSRP modulates melanoma growth and efficacy of vemurafenib. Biochim. Biophys. Acta Gene Regul. Mech..

[44] Olaussen KA (2006). DNA repair by ERCC1 in non-small-cell lung cancer and cisplatin-based adjuvant chemotherapy. N. Engl. J. Med..

[45] Bryant HE (2005). Specifc killing of BRCA2-defcient tumours with inhibitors of poly(ADP-ribose) polymerase. Nature.

[46] Dupré A (2008). A forward chemical genetic screen reveals an inhibitor of the Mre11-Rad50-Nbs1 complex. Nat. Chem. Biol..

[47] Cao J (2017). Targeting of the MAPK and AKT pathways in conjunctival melanoma shows potential synergy. Oncotarget.

[48] Hao S (2018). Transcriptome analysis of phycocyanin-mediated inhibitory functions on non-small cell lung cancer A549 cell growth. Mar. Drugs.

[49] Liu W (2020). Verapamil extends lifespan in *Caenorhabditis elegans* by inhibiting calcineurin activity and promoting autophagy. Aging (Albany NY).

[50] Ma Y (2019). Extracellular vesicles from human umbilical cord mesenchymal stem cells improve nerve regeneration after sciatic nerve transection in rats. J. Cell. Mol. Med..

[51] Yang Q (2020). Effects of the different-sized external stents on vein graft intimal hyperplasia and inflammation. Ann. Transl. Med..

