# Supplementary material for: Fangchinoline suppresses conjunctival melanoma by directly binding FUBP2 and inhibiting the homologous recombination pathway
Source: Cell Death Dis. 2021 Apr 7;12(4):380. doi: 10.1038/s41419-021-03653-4 (PMC8027391; doi:10.1038/s41419-021-03653-4)
Supplement: Supplementary file 1 — Supplementary Figure Legends [file 41419_2021_3653_MOESM1_ESM.docx]

**Supplementary Figure Legends**

**Figure S1. Effects of fangchinoline on liver and kidney damage in CM-AS16 xenograft tumor model.** NCG Mice were treated with increasing doses of fangchinoline 0  mg/kg/d (n=7), 25  mg/kg/d (n=4) and 50  mg/kg/d (n=6) or MEK162 50  mg/kg/d (n=6) for 22 days. Liver fuction (ALT and AST) and renal function (creatinine and urea) were detected by kinetic method. Differences of ALT (A), AST (B), creatinine (C) and urea (D) between different treatment groups were shown.

**Figure S2. Fangchinoline increased the sensitivity of A375 cells to cisplatin on nu/nu nude mice.** (A) Growth curves of A375 xenograft tumors in control mice and mice treated with ciaplatin (2 mg/kg) and/or fangchinoline (10 or 50 mg/kg). Tumor volume was measured once every 3 days. After 14 days, the mice were sacrificed, and the tumors were removed and analyzed. Data represent mean ± SD, n = 6 per group. **P* < 0.05, ***P* < 0.01, *****P* < 0.0001, by two-way ANOVA. (B) To evaluate the effects of fangchinoline and/or cisplatin treatment, *in vivo* apoptosis was evaluated by staining of A375 tumor tissues for Tunel. The sections were counterstained with DAPI. Representative images were shown. Scale bar: 100 μm.

**Figure S3. Fangchinoline decreased the levels of c-Myc compared to controls in A375 tumor tissues.** Immunofluorescence staining of c-Myc in A375 tumor tissues with fangchinoline (0 or 50 mg/kg/d) treatment for 14 days. Representative images were shown. n=3. Scale bar: 100 μm.

**Figure S4. The structures of cepharanthine, fangchinoline, tetrandrine and berbamine hydrochloride.**

**Figure S5. The untrimmed whole western blot images in the manuscript.**

**Figure S6. Fangchinoline suppressed the HR pathway and increased sensitivity to DNA damage-inducing drugs in CM2005.1 cells.** (A) Transcriptional expressions of HR factors *RAD50*, *RAD51*, *BRCA1* and *BRCA2* were decreased in CM2005.1 cells after treatment with 5 μM or 8 μM fangchinoline. n=4. (B) Transcriptional expression of c-Myc was decreased in CM2005.1 cells after treatment with 8 μM fangchinoline. n=4. (C) The protein expressions of c-Myc, RAD51 and BRCA1 were decreased in CM2005.1 cells after treatment with fangchinoline. n=3. (D) Representative images of RAD51 foci induced by cisplatin (80 μM for 24 h) treated with or without 2 μM fangchinoline in CM2005.1 cells [red: RAD51; blue: Hoechst] (left). Scale bar: 5 μm. Fangchinoline significantly decreased the number of RAD51 foci induced by cisplatin in CM2005.1 cells. n=3. (E) Fangchinoline increased the sensitivity of CM2005.1 cells to cisplatin. (F) Fangchinoline increased the sensitivity of CM2005.1 cells to doxorubicin. n=3. (G) The transcriptional expressions of FUBP2, c-Myc, RAD51 and BRCA1 were reduced after treatment with shFUBP2s in CM2005.1 cells. n=4. (H) The protein expressions of c-Myc, RAD51 and BRCA1 were decreased in CM2005.1 cells after treatment with shFUBP2s. n=3. Data represent the mean ± SD. **P* < 0.05, ***P* < 0.01, ****P* < 0.001, by Student’s t-test.

**Figure S7. Fangchinoline suppressed the HR pathway and increased sensitivity to DNA damage-inducing drugs in CRMM1 cells.** (A) Transcriptional expressions of HR factors *RAD50*, *RAD51*, *BRCA1* and *BRCA2* were decreased in CRMM1 cells after treatment with 5 μM or 8 μM fangchinoline. n=4. (B) Transcriptional expression of c-Myc was decreased in CRMM1 cells after treatment with 8 μM fangchinoline. n=4. (C) The protein expressions of c-Myc, RAD51 and BRCA1 were decreased in CRMM1 cells after treatment with fangchinoline. n=3. (D) Representative images of RAD51 foci induced by cisplatin (80 μM for 24 h) treated with or without 2 μM fangchinoline in CRMM1 cells [red: RAD51; blue: Hoechst] (left). Scale bar: 5 μm. Fangchinoline significantly decreased the number of RAD51 foci induced by cisplatin in CRMM1 cells. n=3. (E) Fangchinoline increased the sensitivity of CRMM1 cells to cisplatin. (F) Fangchinoline increased the sensitivity of CRMM1 cells to doxorubicin. n=3. (G) The transcriptional expressions of FUBP2, c-Myc, RAD51 and BRCA1 were reduced after treatment with shFUBP2s in CRMM1 cells. n=4. (H) The protein expressions of c-Myc, RAD51 and BRCA1 were decreased in CRMM1 cells after treatment with shFUBP2s. n=3. Data represent the mean ± SD. **P* < 0.05, ***P* < 0.01, ****P* < 0.001, by Student’s t-test.

**Figure S8. Fangchinoline suppressed the HR pathway and increased sensitivity to DNA damage-inducing drugs in CRMM2 cells.** (A) Transcriptional expressions of HR factors *RAD50*, *RAD51*, *BRCA1* and *BRCA2* were decreased in CRMM2 cells after treatment with 5 μM or 8 μM fangchinoline. n=4. (B) Transcriptional expression of c-Myc was decreased in CRMM2 cells after treatment with 8 μM fangchinoline. n=4. (C) The protein expressions of c-Myc, RAD51 and BRCA1 were decreased in CRMM2 cells after treatment with fangchinoline. n=3. (D) Representative images of RAD51 foci induced by cisplatin (80 μM for 24 h) treated with or without 2 μM fangchinoline in CRMM2 cells [red: RAD51; blue: Hoechst] (left). Scale bar: 5 μm. Fangchinoline significantly decreased the number of RAD51 foci induced by cisplatin in CRMM2 cells. n=3. (E) Fangchinoline increased the sensitivity of CRMM2 cells to cisplatin. (F) Fangchinoline increased the sensitivity of CRMM2 cells to doxorubicin. n=3. (G) The transcriptional expressions of FUBP2, c-Myc, RAD51 and BRCA1 were reduced after treatment with shFUBP2s in CRMM2 cells. n=4. (H) The protein expressions of c-Myc, RAD51 and BRCA1 were decreased in CRMM2 cells after treatment with shFUBP2s. n=3. Data represent the mean ± SD. **P* < 0.05, ***P* < 0.01, ****P* < 0.001, by Student’s t-test.

**Figure S9. Fangchinoline suppressed the HR pathway.** The protein expressions of c-Myc, RAD51 and BRCA1 were decreased after treatment with fangchinoline in A375 (A), K562 (C), A549 (D), ISK (E) and SKOV3 (F) cells or shFUBP2s in A375 cells (B). n=3.

**Figure S10. The untrimmed whole western blot images in the supporting information Figure S6, Figure S7 and Figure S8.**

**Figure S11. The untrimmed whole western blot images in the supporting information Figure S9.**

**Figure S12. The chemical synthesis of positive and negative probes.**
