# Supplementary material for: Fangchinoline suppresses conjunctival melanoma by directly binding FUBP2 and inhibiting the homologous recombination pathway
Source: Cell Death Dis. 2021 Apr 7;12(4):380. doi: 10.1038/s41419-021-03653-4 (PMC8027391; doi:10.1038/s41419-021-03653-4)
Supplement: Supplementary file 2 — Supplementary Chemical Synthesis [file 41419_2021_3653_MOESM2_ESM.docx]

**Supplementary** **Chemical Synthesis of Probes**

As shown in FigS12, fangchinoline (**1-1**) had a free phenol group, which could be coupled with commercially available 3-(but-3-yn-1-yl)-3-(2-iodoethyl)-3H-diazirine (**1-2**) under alkaline condition. After flash chromatography, positive probe was obtained.

Because of the obscurity of structure-activity relationship (SAR) among the benzyl tetrahydroisoquinoline alkaloids, a simple ether-type negative probe (**1-4**) was designed. Considering direct substitution of iodinyl group in **1-2** by methoxyl group might result in formation of alkene or diazirine degradation, we chose another synthetic route based on the synthesis of **1-2** (ref. ^1^). Briefly, reported alcohol **1-5** (ref. ^1^) was transformed into methyl ether **1-6** via deprotonation by sodium hydride and reaction with iodomethane. After deprotection in the acidic condition, the free carbonyl group in **1-7** was transformed into diazirine group by reported three-step procedure^1^, and **1-4** was finally obtained.

*General information*

All solvents and reagents were commercially available and purchased from commercial suppliers such as Adamas-beta®, Bide pharmatech, etc., and directly used without further purification. Ketal **1-5** was synthesized according reported procedures. Flash column chromatography was performed using HSGF 254 (150–200 μm thickness; Yantai Huiyou Co., China). Analytical thin-layer chromatography (TLC) was performed using HSGF 254 (150–200 μm thickness; Yantai Huiyou Co., China), and spots were visualized with UV light, iodine, and potassium permanganate staining. Unless otherwise noted, reactions were performed without protection of inert gas. Reaction conditions and yields were not optimized. Chiral column chromatography was conducted in Daicel Chiral Technologies (China) CO. Nuclear magnetic resonance (NMR) spectroscopy was performed on a Bruker AMX-400 NMR (IS as TMS). Chemical shifts were reported in parts per million (ppm, *δ*) downfield from tetramethylsilane. Proton coupling patterns were described as singlet (s), doublet (d), triplet (t), quartet (q), multiplet (m), and broad (br). High-resolution mass spectra (HRMS) was obtained by electrospray ionization (ESI) using a Waters GCT Premie and Waters LCT.

*Synthesis of 7-(3-(but-3-yn-1-yl)-3H-diazirin-3-yl)-fangchinoline (****1-3****)*

Fangchinoline (**1-1**, 120 mg, 0.2 mmol) and 3-(but-3-yn-1-yl)-3-(2-iodoethyl)-3*H*-diazirine (**1-2**, 55 mg, 0.22 mmol) were dissolved into dry *N*,*N*-dimethylformamide (2 ml) in the nitrogen atmosphere. Potassium carbonate powder (33 mg, 0.24 mmol) was added and the mixture was stirred at 40 °C in the darkness. After 48 h, the mixture was cooled to rt., and diluted with water (20 ml) and extracted three times with dichloromethane (75 ml in total). The organic phase was washed with water (75 ml) and brine (75 ml), then dried with anhydrous sodium sulfate. The solvent was evaporated under reduced pressure and the residue was purified via silica gel column chromatography (dichloromethane/methanol) to get compound **1-3** (36 mg, 25% yield).

^1^H NMR (400 MHz, Chloroform-*d*) *δ* 7.38 (dd, *J*=8.1, 2.2 Hz, 1H), 7.19–7.08 (m, 1H), 6.87 (d, *J*=7.2 Hz, 1H), 6.82 (dd, *J*=8.3, 2.5 Hz, 1H), 6.54 (s, 1H), 6.49 (s, 1H), 6.31 (m, 2H), 5.94 (s, 1H), 3.93 (s, 3H), 3.87 (m, 1H), 3.77 (s, 3H), 3.55 (m, 2H), 3.41 (m, 5H), 3.23 (m, 1H), 3.01–2.75 (m, 7H), 2.67 (s, 3H), 2.35 (s, 3H), 2.02–1.86 (m, 3H), 1.54 (m, 3H), 1.04 (t, *J*=7.0 Hz, 2H); HRMS (ESI) m/z calcd. for C_44_H_49_N_4_O_6_ [M+H]^+^ 729.3652, found 729.3654.

*Synthesis of 2-(but-3-yn-1-yl)-2-(2-methoxyethyl)-1,3-dioxolane (****1-6****)*

2-(2-(But-3-yn-1-yl)-1,3-dioxolan-2-yl)ethan-1-ol (**1-5**, 0.85 g, 5 mmol) was dissolved into dry *N*,*N*-dimethylformamide (25 ml) in the nitrogen atmosphere. At 0 °C sodium hydride (60%, 60% dispersion in mineral oil) was added to the solution (0.3 g, 7.5 mmol). The mixture was stirred at rt. for 30 min, then methyl iodide (0.6 ml, 10 mmol) was injected into mixture via syringe and the reaction was stirred at rt. for 24 h in the darkness. 10% aqueous sodium hydroxide (10 ml) was slowly injected into mixture, and the mixture was poured into 150 ml water and extracted three times with petroleum ether/ethyl acetate (1:1, 150 ml in total). The organic phase was washed with brine (150 ml) and dried with anhydrous sodium sulfate. The solvent was evaporated under reduced pressure and the residue was purified via silica gel column chromatography (petroleum ether/ethyl acetate) to get compound **1-6** (0.7 g, 76% yield).

^1^H NMR (400 MHz, Chloroform-*d*) *δ* 3.94 (s, 4H), 3.46 (t, *J*=6.9 Hz, 2H), 3.32 (s, 3H), 2.27 (ddd, *J* = 8.6, 6.8, 2.7 Hz, 1H), 1.97–1.86 (m, 5H).

*Synthesis of 1-methoxyhept-6-yn-3-one (****1-7****)*

Compound **1-6** (0.7 g, 3.8 mmol) was dissolved into acetone (11 ml) and *p*-toluenesulfonic acid monohydrate (0.18 g 0.95 mmol) was added. The reaction was stirred at rt. for 2 h, then poured into saturated aqueous sodium bicarbonate (20 ml) and extracted with three times ethyl acetate (60 ml in total). The organic phase was washed with brine (60 ml) and dried with anhydrous sodium sulfate. The solvent was evaporated under reduced pressure and the residue was purified via silica gel column chromatography (petroleum ether/ethyl acetate) to get compound **1-7** (0.51 g, 76% yield).

^1^H NMR (400 MHz, Chloroform-*d*) *δ* 3.65 (t, *J*=6.2 Hz, 2H), 3.33 (s, 3H), 2.74–2.69 (m, 2H), 2.67 (t, *J*=6.2 Hz, 2H), 2.45 (td, *J*=7.3, 2.7 Hz, 2H), 1.94 (t, *J*=2.7 Hz, 2H).

*Synthesis of 3-(but-3-yn-1-yl)-3-(2-methoxyethyl)-3H-diazirine (****1-4****)*

Compound **1-7** (140 mg, 1 mmol) was dissolved into ammonia/methanol solution (7 M, 1 ml) in the nitrogen atmosphere. The mixture was stirred at 0 °C for 24 h, then hydroxylamine-*O*-sulfonic acid (130 mg, 1.2 mmol) and dry methanol (2 ml) were added at 0 °C to the solution. The mixture was stirred at rt. for 12 h, then the slurry was filtered with celite and the solid residue was washed with methanol. The filtrate was collected and evaporated under reduced pressure. The residue was dissolved into dichloromethane (2 ml) and treated with triethylamine (175 μL, 1.26 mmol). Iodine (300 mg, 1.2 mmol) was slowly added at 0 °C and the solution was stirred at rt. for 10 min. The solvent was evaporated under reduced pressure and the residue was purified via silica gel column chromatography (petroleum ether/ethyl acetate) to get compound **1-4** (35 mg, 23% yield).

^1^H NMR (400 MHz, Chloroform-*d*) *δ* 3.30 (s, 3H), 3.20 (t, *J*=6.3 Hz, 2H), 2.02 (td, *J*=7.5, 2.6 Hz, 2H), 1.97 (t, *J*=2.6 Hz, 1H), 1.72–1.64 (m, 4H); HRMS (ESI) m/z calcd. for C_8_H_12_N_2_O [M+H]^+^ 152.0950, found 152.0952.

**References**

1. Li, Z., Hao, P., Li, L., Tan, C. Y. J., Cheng, X., Chen, G. Y. J. et al. Design and Synthesis of Minimalist Terminal Alkyne-Containing Diazirine Photo-Crosslinkers and Their Incorporation into Kinase Inhibitors for Cell- and Tissue-Based Proteome Profiling. *Angew. Chem. Int. Ed. Engl.* **52**, 8551–8556 (2013).
