# Supplementary material for: Fangchinoline suppresses conjunctival melanoma by directly binding FUBP2 and inhibiting the homologous recombination pathway
Source: Cell Death Dis. 2021 Apr 7;12(4):380. doi: 10.1038/s41419-021-03653-4 (PMC8027391; doi:10.1038/s41419-021-03653-4)
Supplement: Supplementary file 3 — Supplementary Information [file 41419_2021_3653_MOESM3_ESM.docx]

**Supplementary Materials and Methods**

**Primer sequences for qRT-PCR:**

*GAPDH*: Forward 5’- AAGGTGAAGGTCGGAGTCAAC-3’,

Reverse 5’- GGGGTCATTGATGGCAACAATA-3’;

*β-Actin*: Forward 5’-CTGGAACGGTGAAGGTGACA-3’,

Reverse 5’-AAGGGACTTCCTGTAACAATGCA-3’;

*FUBP2*: Forward 5’- CAAGCCTGTGAGATGGTGATG-3’,

Reverse 5’-GCTCCCGTCTGCTGGTTTAT-3’;

*c-Myc*: Forward 5’-GGACCCGCTTCTCTGAAAG-3’,

Reverse 5’-GTCGAGGTCATAGTTCCTGTTG-3’;

*RAD50*: Forward 5’-GATCGAACGTCATCTTGCTTAC-3’,

Reverse 5’-ATGTCAAGTTCTGCCATAGACA-3’;

*RAD51*: Forward 5’-GCCCTTTACAGAACAGACTACT-3’,

Reverse 5’-TTGAGCTACCACCTGATTAGTG-3’;

*BRCA1*: Forward 5’-GCCAAAGTAGCTGATGTATTGG-3’,

Reverse 5’-CAGTTACATGGCTTAAGTTGGG-3’;

*BRCA2*: Forward 5’-TAAGAGGAAGGACCGAGTAGAA-3’,

Reverse 5’-TGATCCATCATCTGGTACATGG-3’.
