# Supplementary material for: Fangchinoline suppresses conjunctival melanoma by directly binding FUBP2 and inhibiting the homologous recombination pathway
Source: Cell Death Dis. 2021 Apr 7;12(4):380. doi: 10.1038/s41419-021-03653-4 (PMC8027391; doi:10.1038/s41419-021-03653-4)
Supplement: Supplementary file 4 — Supplementary Table 1 [file 41419_2021_3653_MOESM4_ESM.docx]

**Supplementary Table 1.** The anti-CM activity of three cepharanthine analogues (fangchinoline, tetrandrine and berbamine hydrochloride). n=3.

| **IC_50_ (μM)** | **CM-AS16** | **CRMM2** | **CRMM1** | **CM2005.1** | **MRC5** | **MRC5/**  **CM-AS16** |
| --- | --- | --- | --- | --- | --- | --- |
| Cepharanthine | 1.63 ± 0.24 | 0.792 ± 0.013 | 4.69 ± 0.17 | 7.44 ± 0.08 | 8.44 ± 0.54 | 5.18 |
| Fangchinoline | 5.67 ± 0.17 | 3.60 ± 0.05 | 2.68 ± 0.07 | 7.40 ± 0.45 | 4.35 ± 0.11 | 0.77 |
| Tetrandrine | 3.20 ± 0.06 | 2.16 ± 0.02 | 4.77 ± 0.24 | 9.70 ± 0.07 | 5.75 ± 0.11 | 1.80 |
| Berbamine Hydrochloride | 8.46 ± 0.22 | 4.14 ± 0.50 | 8.16 ± 0.13 | 20.20 ± 0.49 | 9.03 ± 0.22 | 1.07 |
| Fangchinoline Probe | 0.95 ± 0.08 | 4.26 ± 0.09 | 1.90 ± 0.09 | 3.68 ± 0.07 | 3.23 ± 0.15 | 3.44 |
| MEK162 | 0.0077 ± 0.0016 | 0.0136 ± 0.0006 | >50 | 0.068 ± 0.008 | 6.32 ± 0.16 | 820.78 |
