# Supplementary material for: Fangchinoline suppresses conjunctival melanoma by directly binding FUBP2 and inhibiting the homologous recombination pathway
Source: Cell Death Dis. 2021 Apr 7;12(4):380. doi: 10.1038/s41419-021-03653-4 (PMC8027391; doi:10.1038/s41419-021-03653-4)
Supplement: Supplementary file 5 — Supplementary Table 2 [file 41419_2021_3653_MOESM5_ESM.docx]

**Supplementary Table 2.** Nine proteins were identified as potential targets that were specifically bound to fangchinoline in ABPP assay.

| **Protein Group** | **-10lgP** | **Coverage**  **(%)** | **#Peptides** | **#Unique** | **Avg. Mass** | **Description** |
| --- | --- | --- | --- | --- | --- | --- |
| 57 | 279.06 | 6 | 22 | 15 | 553139 | Epiplakin |
| 123 | 184.1 | 16 | 11 | 11 | 104779 | Hypoxia up-regulated protein 1 |
| 198 | 165.45 | 12 | 6 | 6 | 78606 | Heat shock 70 kDa protein 4 |
| 158 | 115.6 | 13 | 7 | 7 | 73028 | Far upstream element-binding protein 2 |
| 401 | 81.76 | 9 | 2 | 2 | 36269 | Pro-cathepsin H |
| 285 | 74.43 | 31 | 4 | 4 | 13303 | Selenoprotein H |
| 631 | 51.56 | 4 | 1 | 1 | 24458 | Ribosomal RNA small subunit methyltransferase NEP1 |
| 524 | 41.45 | 3 | 1 | 1 | 31983 | Immunoglobulin heavy constant gamma 1 |
| 251 | 38.69 | 0 | 1 | 1 | 3013943 | Titin |
