# Supplementary figures and images for: Fangchinoline suppresses conjunctival melanoma by directly binding FUBP2 and inhibiting the homologous recombination pathway
Source: Cell Death Dis. 2021 Apr 7;12(4):380. doi: 10.1038/s41419-021-03653-4 (PMC8027391; doi:10.1038/s41419-021-03653-4)

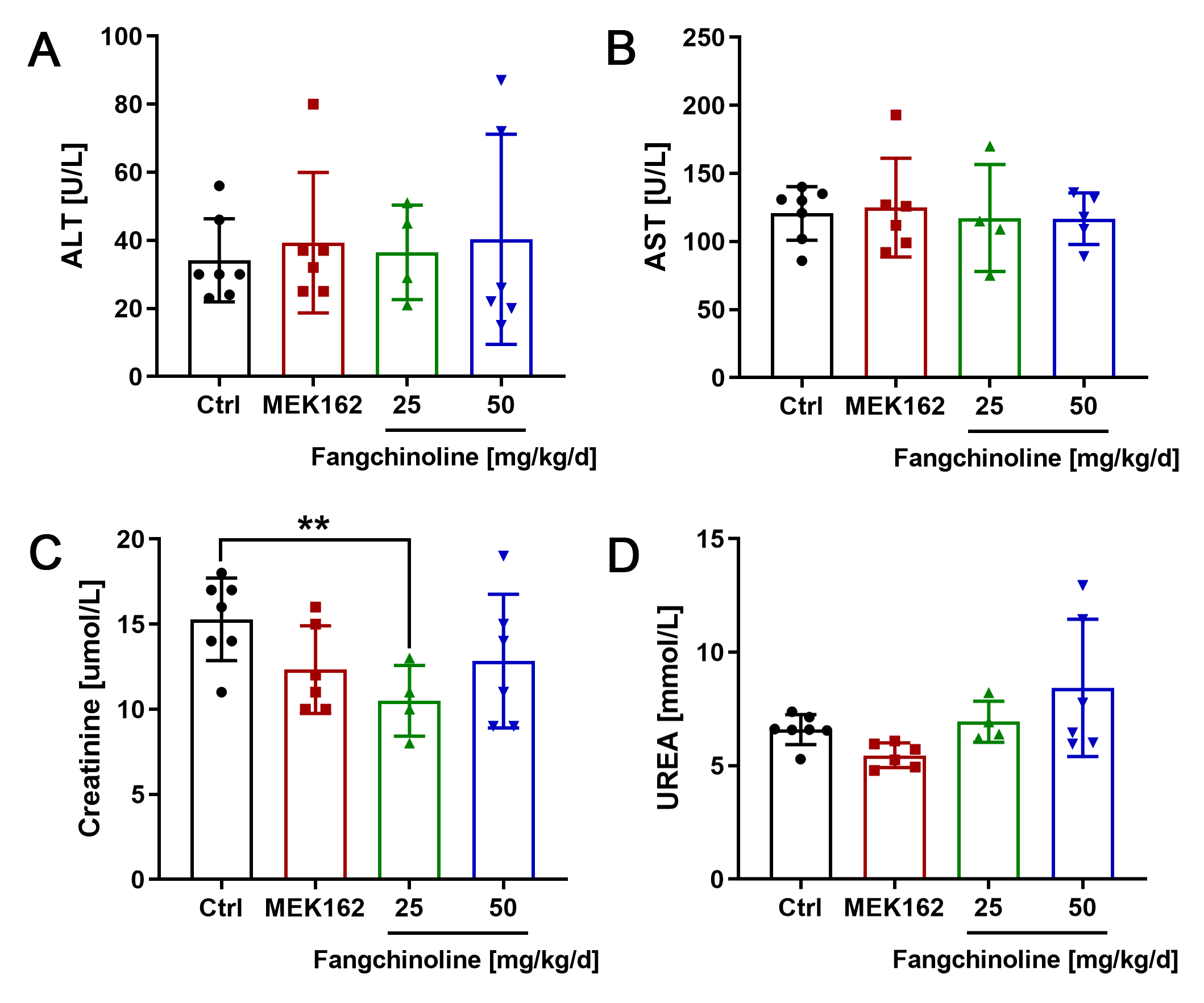

Supplement: Supplementary file 6 — Figure S1. Effects of fangchinoline on liver and kidney damage in CM-AS16 xenograft tumor model. [file 41419_2021_3653_MOESM6_ESM.tif]

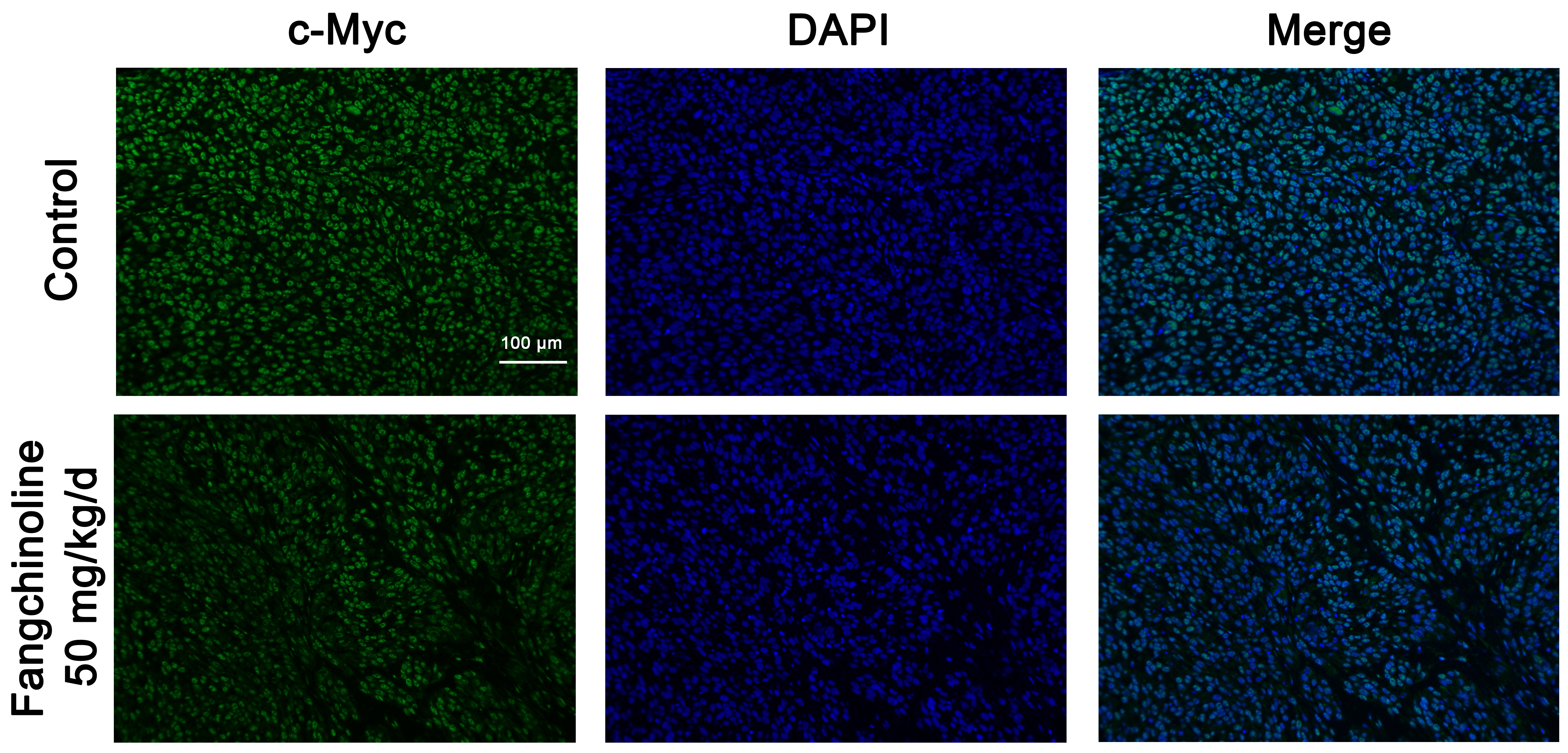

Supplement: Supplementary file 8 — Figure S3. Fangchinoline decreased the levels of c-Myc compared to controls in A375 tumor tissues. [file 41419_2021_3653_MOESM8_ESM.tif]

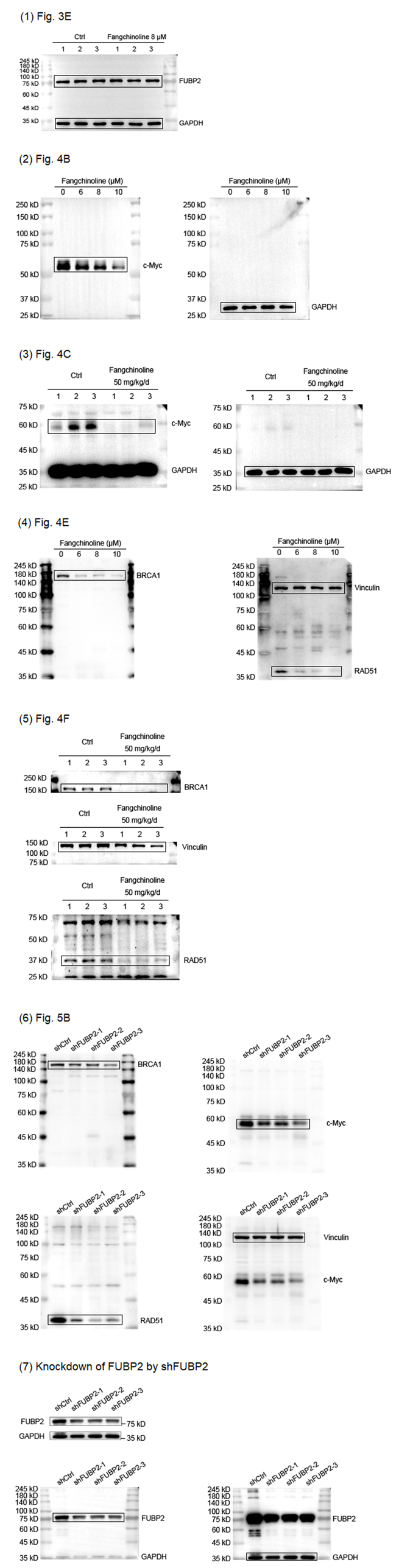

Supplement: Supplementary file 10 — Figure S5. The untrimmed whole western blot images in the manuscript. [file 41419_2021_3653_MOESM10_ESM.tif]

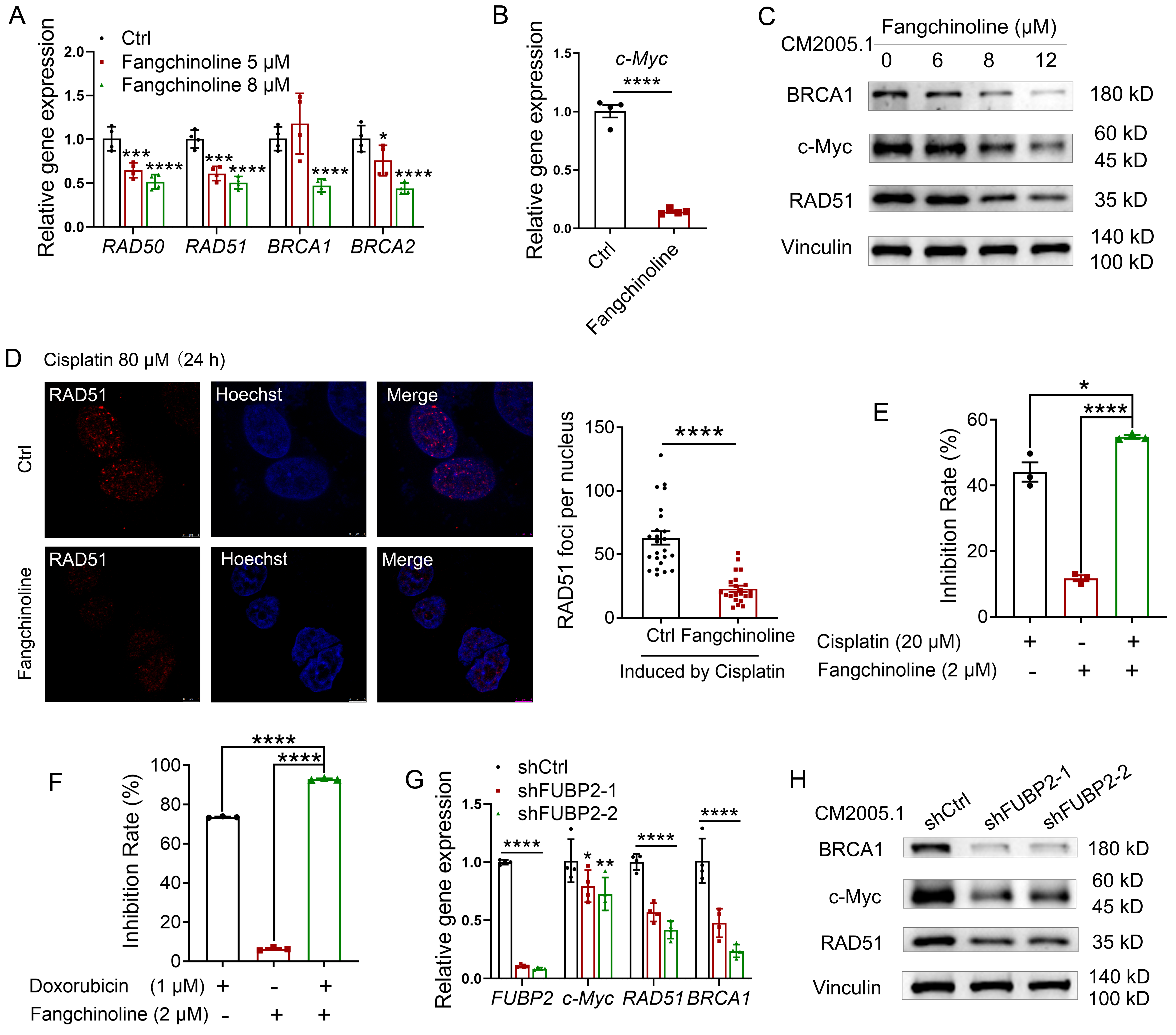

Supplement: Supplementary file 11 — Figure S6. Fangchinoline suppressed the HR pathway and increased sensitivity to DNA damage-inducing drugs in CM2005.1 cells. [file 41419_2021_3653_MOESM11_ESM.tif]

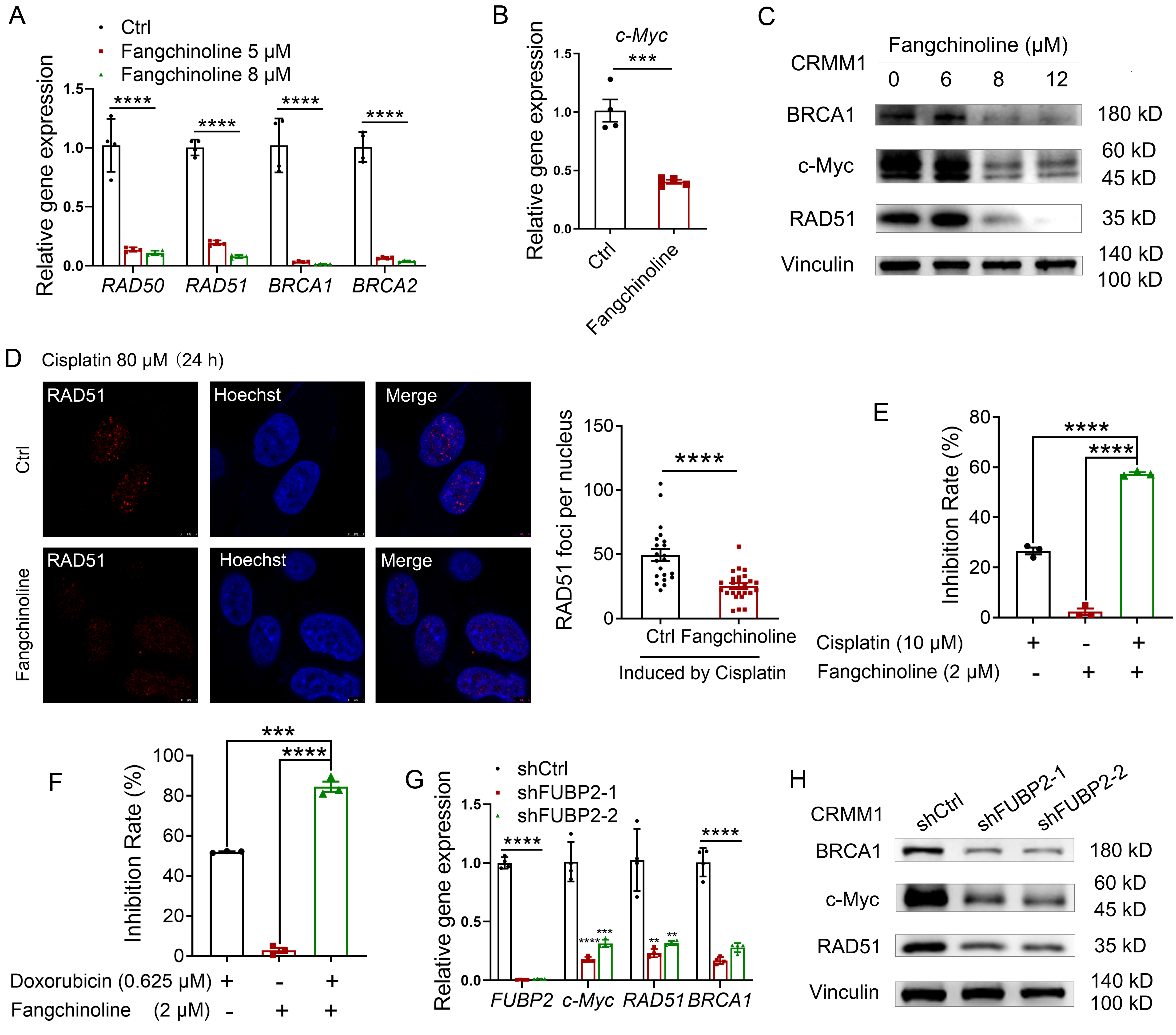

Supplement: Supplementary file 12 — Figure S7. Fangchinoline suppressed the HR pathway and increased sensitivity to DNA damage-inducing drugs in CRMM1 cells. [file 41419_2021_3653_MOESM12_ESM.tif]

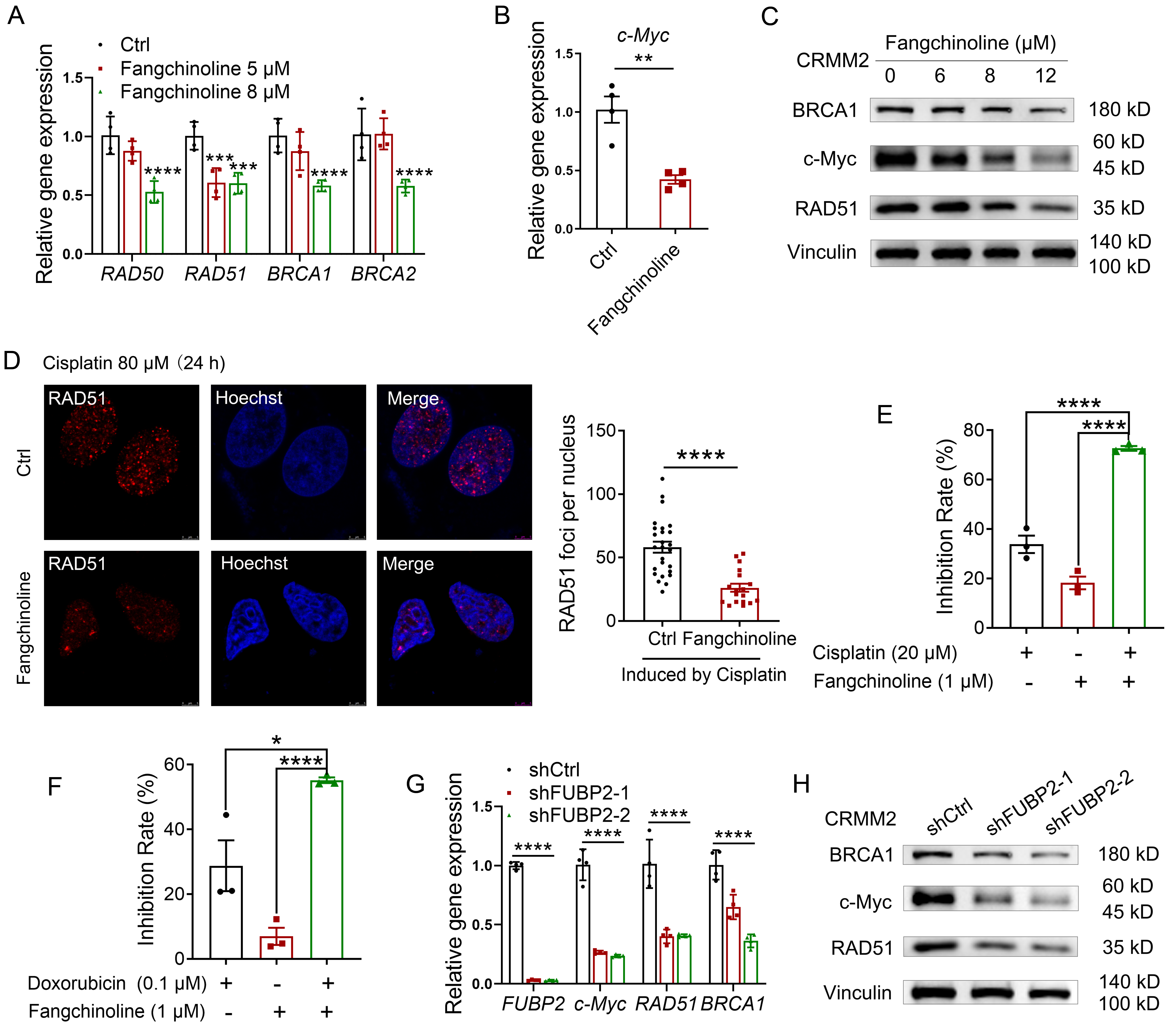

Supplement: Supplementary file 13 — Figure S8. Fangchinoline suppressed the HR pathway and increased sensitivity to DNA damage-inducing drugs in CRMM2 cells. [file 41419_2021_3653_MOESM13_ESM.tif]

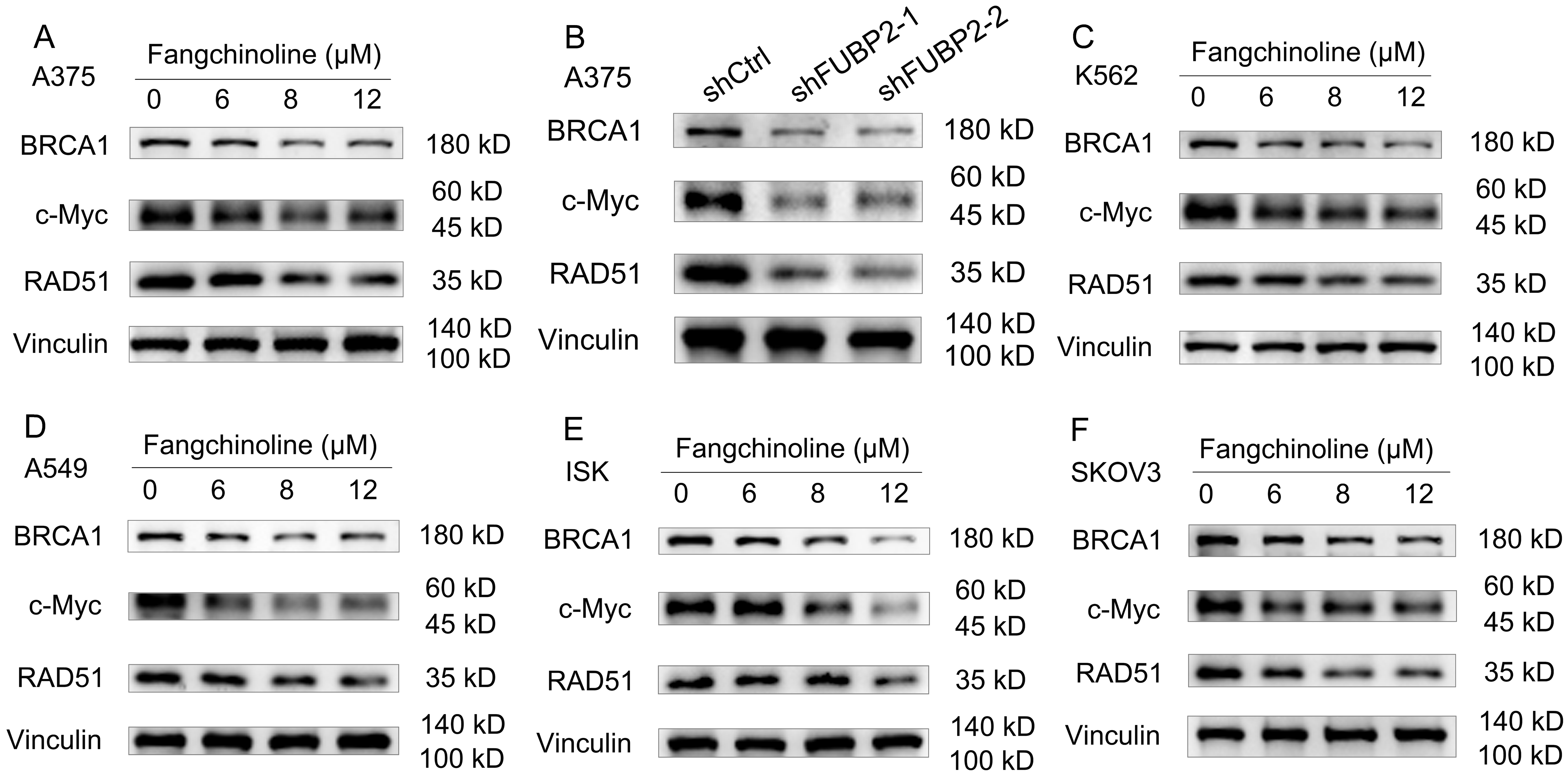

Supplement: Supplementary file 14 — Figure S9. Fangchinoline suppressed the HR pathway. [file 41419_2021_3653_MOESM14_ESM.tif]
